# Adjustment for survey non‐representativeness using record‐linkage: refined estimates of alcohol consumption by deprivation in Scotland

**DOI:** 10.1111/add.13797

**Published:** 2017-04-25

**Authors:** Emma Gorman, Alastair H. Leyland, Gerry McCartney, Srinivasa Vittal Katikireddi, Lisa Rutherford, Lesley Graham, Mark Robinson, Linsay Gray

**Affiliations:** ^1^ MRC/CSO Social and Public Health Sciences Unit, University of Glasgow Glasgow UK; ^2^ Department of Economics Lancaster University Lancaster UK; ^3^ NHS Health Scotland Glasgow UK; ^4^ ScotCen Social Research Edinburgh UK; ^5^ Information Services Division NHS National Services Scotland Edinburgh UK; ^6^ Department of Epidemiology, Mailman School of Public Health Columbia University New York NY USA

**Keywords:** Alcohol consumption, alcohol‐related harm, bias, epidemiology, health surveys, non‐participation, record‐linkage, Scotland

## Abstract

**Background and aims:**

Analytical approaches to addressing survey non‐participation bias typically use only demographic information to improve estimates. We applied a novel methodology which uses health information from data linkage to adjust for non‐representativeness. We illustrate the method by presenting adjusted alcohol consumption estimates for Scotland.

**Design:**

Data on consenting respondents to the Scottish Health Surveys (SHeSs) 1995–2010 were linked confidentially to routinely collected hospital admission and mortality records. Synthetic observations representing non‐respondents were created using general population data. Multiple imputation was performed to compute adjusted alcohol estimates given a range of assumptions about the missing data. Adjusted estimates of mean weekly consumption were additionally calibrated to per‐capita alcohol sales data.

**Setting:**

Scotland.

**Participants:**

13 936 male and 18 021 female respondents to the SHeSs 1995–2010, aged 20–64 years.

**Measurements:**

Weekly alcohol consumption, non‐, binge‐ and problem‐drinking.

**Findings:**

Initial adjustment for non‐response resulted in estimates of mean weekly consumption that were elevated by up to 17.8% [26.5 units (18.6–34.4)] compared with corrections based solely on socio‐demographic data [22.5 (17.7–27.3)]; other drinking behaviour estimates were little changed. Under more extreme assumptions the overall difference was up to 53%, and calibrating to sales estimates resulted in up to 88% difference. Increases were especially pronounced among males in deprived areas.

**Conclusions:**

The use of routinely collected health data to reduce bias arising from survey non‐response resulted in higher alcohol consumption estimates among working‐age males in Scotland, with less impact for females. This new method of bias reduction can be generalized to other surveys to improve estimates of alternative harmful behaviours.

## Introduction

Accurate data on addictive substance use are necessary to inform policy development, implementation and evaluation 1, 2 and for alcohol research. In many countries, estimates of population consumption of legal addictive substances are derived from taxation or sales data 3. However, data on trends in alcohol consumption by social and demographic groups (such as age, gender and socio‐economic position) and the pattern in which substances are consumed (for example, frequency and amount per occasion) typically require the administration of population‐sampled surveys.

In the Scottish context, the Scottish Health Surveys (SHeS) 4 offer detailed exploration of alcohol consumption at the individual level. Although providing important additional insights compared to sales data 5, survey‐derived estimates face various biases—including those arising from non‐participation of invited respondents (unit non‐response), social desirability and recall 6—which may threaten internal validity and generalizability to the population. Additionally, sampling frames of many population‐sampled health surveys, including the SHeSs, are confined to private residences, excluding institutionalized and transient populations. Furthermore, incidence rates of alcohol‐related harm 7, 8, as well as all‐cause mortality 8, 9, have been found to be substantially lower among survey respondents compared with the general population, as we identified from record‐linkage in SHeS 10. These features contribute to underestimation of population consumption from surveys 11, 12, 13, 14, evident as a substantial differential between survey‐ and sales data‐based estimates of mean weekly units consumed 15, 16, 17, thus hampering alcohol research.

Understanding the consequences of survey non‐response is particularly salient, as survey response levels are declining 18, 19. Poor health and risky health behaviours correlate with non‐response 20, 21, 22, suggesting that the estimation of health behaviour prevalence could be biased. Adjustments for non‐response are often confined to a limited set of socio‐demographic variables (as is the case in the SHeSs 4), with the use of survey weighting being the most commonly adopted method. This is intended to align the socio‐demographic profile of the survey to that of the target population, but any further differences between respondents and non‐respondents within socio‐demographic groups are not corrected, so weights are likely to be mis‐specified. The addition of health measures may be further informative, although there have been few attempts 23, 24, 25 to incorporate these, not least because the necessary data—comparable across respondents and non‐respondents—are not readily available. However, it is possible for equivalent information to be obtained directly via record‐linkage for those who have responded and implicitly for those who have not 10, 26, which is the case for SHeS.

Even with a broader range of informative data, approaches to adjust for non‐participation necessarily rely upon untestable assumptions about the nature of the missing data. Presenting results based on only one set of assumptions may convey an unrealistic level of certainty about the estimates. Sensitivity analyses based on a range of credible assumptions recognize this uncertainty. Information from additional sources about the probable behaviour of non‐respondents 14 can be used to establish plausible scenarios, with each forming the basis of inference, offering a range of informative estimates for data users to consider.

The aims of this study were threefold:

- Exploit linkage of survey records to administrative health data to adjust for health‐related non‐representativeness in alcohol consumption estimates;
- Conduct sensitivity analysis given a range of assumptions about the unobserved data; and
- Triangulate adjusted survey estimates with alcohol sales data.

In so doing, we quantify the impact of survey non‐response on population estimates and socio‐economic inequalities in alcohol consumption, using generalizable methodology.

## Methods

### Data

#### Baseline survey and population data

The SHeSs are a series of stratified, cluster‐sampled repeated cross‐sectional surveys designed to describe the health of the Scottish population living in private households 26. We used the surveys conducted in 1995 27, 1998 28, 2003 29, 2008 30, 2009 31 and 2010 4, henceforth ‘baseline years’ (adult response percentages ranged from 55% in 2010 to 84% in 1995 (Table 1)). Analyses were restricted to data on individuals aged 20–64 years, as this age range was available in all survey years and enhanced comparability between the survey sampling frame and population data. Survey weights, which had been constructed previously to account for the survey sampling design, incorporating differential selection of addresses/households, calibration to match population estimates for age/sex and health board and within‐household response, were used throughout 32. The alcohol measures of interest were: usual weekly alcohol consumption derived using the quantity–frequency method 33; the prevalence of non‐drinkers; binge drinking (consumption in excess of 6/8 units (1 alcohol unit is measured as 10 ml or 8 g of pure alcohol) on the heaviest drinking day in the last 7 days for women/men) 29 and potential problem‐drinking—defined as two or more positive answers on the CAGE (Cut‐down, Annoyed, Guilt, Eye‐opener) instrument 34, 35.

**Table 1:** Response proportions and consent to linkage in the Scottish Health Surveys, 1995–2010.

Survey year	Household response proportion, %	Adult response proportion, %	Proportion consenting to linkage, %	No. of men aged 20–64 years	No. of women aged 20–64 years
1995	81	84	93	3118	3867
1998	77	76	92	2944	3674
2003	67	60	91	2353	3028
2008	61	54	86	1683	2234
2009	64	56	85	1944	2647
2010	63	55	86	1894	2571
Total	69	64	89	13 936	18 021

General population data comparable with each SHeS survey were constructed using mid‐year population estimates for small‐area geographical units (i.e. datazones which contain approximately 350 households and populations of 500–1000 residents 36) from the National Records of Scotland (NRS) by sex and 5‐year age group at each baseline year 37. Datazone‐level population count estimates were not available for mid‐1995, so mid‐1996 estimates were used.

Area‐based measures of deprivation were matched to both the survey records and population data. The Carstairs 2001 measure of small‐area material disadvantage was used in the 1995 and 1998 baseline years, and the Scottish Index of Multiple Deprivation (SIMD) 38 from 2003 onwards 39.

#### Morbidity and mortality records

The Scottish Morbidity Records (SMR) are hospital episode statistics drawn from routinely collected NHS records of socio‐demographic, episode management and clinical data across Scotland 40 and have been found to be ~90% accurate in recording the correct diagnosis 41 and ~99% complete 42. In‐patient and day cases discharged from general and mental health specialities with an alcohol‐related diagnosis in any diagnostic position 43 and mortality data collected by NRS were classified using the International Classification of Disease (ICD) 9th and 10th editions. For consenting SHeS respondents [range of 85% in 2009 to 93% in 1995 (Table 1)], SMR and NRS records were linked to survey records. Morbidity and mortality data were available until the end of 2011, allowing a maximum follow‐up period of 16 years from 1995. Two overlapping binary measures (collectively referred to as ‘harm’) were created: death due to any cause and any alcohol‐related event (hospitalization or the primary cause of death 44). Data associated with baseline years 2008, 2009 and 2010 were pooled to accommodate smaller sample sizes of the annual format surveys and shorter follow‐up periods (Table 1).

#### Sales data

The alcohol sales data used were collected by market research specialists Nielsen/CGA. Estimates of annual sales in Scotland, including both on‐ and off‐trade, were available for all the baseline years except 1998, which were interpolated linearly 45.

### Statistical methodology

We developed a new methodology, detailed elsewhere 46, to correct the non‐participation aspect of bias arising in survey data. Rather than adjust the weighting, we took an imputation approach. Multiple imputation is a statistical technique for analysing incomplete data sets 47, which has the advantage of allowing the flexibility afforded by being amenable to sensitivity analyses 48. In essence, in the absence of data on individual non‐respondents we used comparisons of the composition of survey respondents in terms of age, sex, deprivation and harms with that of the general population to identify the numbers of missing survey respondents within each socio‐demographic/harm combination group. We then created ‘observations’ for non‐responders within each group and imputed their unknown alcohol consumption estimates. We allowed associations between consumption and harm to differ between respondents and non‐respondents.

Our methodology considers the nature of missingness with reference to the classification of missing data mechanisms 47, as follows: data can be missing at random (MAR) or missing not at random (MNAR) 47. MAR is the case where the probability of missingness is unrelated to the unobserved data taking account of the observed data. Alternatively, if the missingness depends upon unobserved data (even after taking account all the information in the observed data), the observations are MNAR. Note that data which are MNAR can become MAR if additional variables are observed and used in analysis; this is a feature of our approach.

Briefly, the approach involves establishing: (1) the total number of missing respondents, based on the ‘effective response level’—the percentage of the sample that both responded to the survey and consented to linkage—and number of observed respondents; (2) the respondent composition in terms of age, sex, deprivation quintile and harms during follow‐up; (3) the population composition; (4) the number of missing respondents within each socio‐demographic–harm combination group by comparison of survey and population data (from steps 2 and 3); (5) creation of synthetic observations for the non‐respondents; (6) conduct of multiple imputation 49 to provide estimates of alcohol consumption measures in the synthetic ‘non‐respondents’, given the data on age, sex, deprivation and harms, and based on the assumption that the consumption data are MAR (Supporting information, Appendix S1); (7) generation of non‐response‐corrected alcohol estimates under the MAR assumption by combining the observed alcohol data on the respondents, and the imputed alcohol data on the synthetic non‐respondents; and (8) altering the MAR imputation model by specific estimates for the mean difference in alcohol consumption between respondents and non‐respondents in sensitivity analyses, allowing for the possibility that the consumption data could be MNAR (Supporting information, Appendix S2). The effects of a range of MNAR scenarios were explored, as outlined in the MNAR sensitivity analyses section below and Supporting information, Appendix S2.

#### MNAR sensitivity analyses

We relaxed the MAR assumption in sensitivity analyses by modifying the imputation model using a pattern‐mixture approach as detailed in Supporting information, Appendix S2 50. This involved changing the imputation model to reflect potential differences in the distribution of alcohol consumption between respondents and non‐respondents given the observed data. This required specifying a value for the mean difference in alcohol consumption between respondents and non‐respondents, after adjusting for observed covariates (this is zero under MAR). The value of this parameter can be varied to represent different assumptions and the impact on substantive conclusions assessed. Two scenarios were considered.

The first MNAR scenario (MNAR^CR^) drew upon data on the number of attempts to contact a household for interview and ‘continuum of resistance’ theory: non‐respondents may be similar to late respondents, as late respondents would have been non‐respondents if efforts to contact them had ceased earlier 14, 51. The specific scenario considered was that the deviation from MAR could be up to twice the adjusted differences in mean consumption between early (≤ 3 attempts to contact) and late respondents (> 3 attempts).

The second MNAR scenario aimed to incorporate the possibility of a subgroup of very heavy drinkers—we focused upon those experiencing harm—whose consumption may not look similar to any observed subgroup and are not ‘adjusted for’ in typical corrections. As the most extreme scenario (*MNAR* ^***^), the imputation model was altered such that sex‐specific mean consumption among non‐respondents experiencing harm was six times greater than the observed mean. This was informed by data on patients with serious alcohol problems hospitalized or in treatment in two Edinburgh hospitals, which estimated mean weekly consumption among this sample as 197.7 units 52. This approach resulted in an adjusted mean among drinkers experiencing harm of 197.5 units, compared with a MAR estimate of 48.4 units in this group. More moderate scenarios were considered, where sex‐specific mean consumption among those experiencing harm was double (*MNAR* ^*^) and quadruple (*MNAR* ^**^) that of their observed counterparts.

#### Sales data‐based triangulation

We adjusted the survey estimates informed by comparison with per capita estimates 53, 54, 55 (Supporting information, Appendix S3). Each overall non‐response adjusted estimate of mean weekly consumption was compared with the per capita consumption estimate for Scotland to assess the magnitude of the remaining coverage gap: that which is not explained by participation bias in our data. This proportionate difference was used to shift up each of the estimates of mean consumption in sex‐ and deprivation quintile‐specific subgroups. All analyses were conducted in Stata/SE version 13.1 (StataCorp LP, College Station, TX, USA).

## Results

The generation of synthetic non‐respondent observations aligned survey and population in terms of sex‐ and area deprivation quintile‐specific percentage breakdowns (Supporting information, Table S1). The differential gradient in the probability of prospective alcohol‐related harm between the survey and population data was corrected in the adjusted data (Supporting information, Table S2).

The MAR adjustment resulted in elevated estimates of weekly consumption among males (Table 2), for whom the magnitude of correction ranged from 1.9% in 1995 to 3.8% in 1998, 2.7% in 2003 and 1.6% in 2008/10, compared with little correction among females. Taking 2003 as an example, in the second scenario MNAR adjustments increased weekly units consumed among males from the original (R) estimate of 21.8 units to 24.6 units (14% increase) in the weakest scenario (MNAR*), and to 33.3 units in the most extreme (53% increase: MNAR***; Table 2). The first scenario MNAR‐based estimates (MNAR^CR^) were generally around those of the second scenario MNAR*. The set of estimates calibrated to sales data ranged from 33.2 to 36.4 units, with the biggest increase of 88% during 2008‐10 (Table 2).

**Table 2:** Mean weekly alcohol consumption estimates for Scottish Health Surveys 1995, 1998, 2003 and 2008–2010 among individuals aged 20–64 years by sex.

Baseline year	1995		1998		2003		2008–10	
	Mean	(95 % CI)/SD	Mean	(95 % CI)/SD	Mean	(95 % CI)/SD	Mean	(95 % CI)/SD
Males								
R	20.8	(19.7–22.0)	20.0	(19.0–21.0)	21.8	(20.5–23.1)	18.8	(17.9–19.6)
MAR	21.2	(20.0–22.4)	20.8	(19.5–22.0)	22.4	(20.3–24.4)	19.1	(18.2–20.1)
MNAR^CR^	22.6	(21.4–23.8)	22.2	(20.9–23.6)	24.9	(22.8–27.0)	20.7	(19.7–21.7)
MNAR^a^	22.8	(21.5 ‐ 24.1)	22.3	(20.9–23.6)	24.6	(22.4–26.7)	20.1	(19.1–21.1)
MNAR^b^	25.9	(24.2 ‐ 27.7)	25.3	(23.5–27.0)	28.9	(26.4–31.5)	22.1	(20.9–23.3)
MNAR^c^	29.1	(26.7 ‐ 31.4)	28.2	(26.0–30.5)	33.3	(30.1–36.5)	24.1	(22.6–25.6)
Calibrated	33.8	*39.3*	34.6	*40.4*	33.2	*41.1*	33.5	*39.9*
Calibrated^a^	34.1	*40.4*	34.9	*41.8*	34.0	*42.3*	33.9	*41.5*
Calibrated^b^	34.7	*43.9*	35.4	*46.1*	35.3	*47.2*	34.6	*45.3*
Calibrated^c^	35.1	*47.0*	35.8	*49.9*	36.4	*51.5*	35.3	*48.8*
Females								
R	6.3	(5.8–6.7)	7.0	(6.6–7.3)	10.8	(10.1–11.6)	8.8	(8.5–9.1)
MAR	6.4	(5.9–6.9)	7.0	(6.5–7.5)	10.8	(9.8–11.7)	8.8	(8.5–9.1)
MNAR^CR^	6.7	(6.2–7.2)	7.3	(6.9–7.8)	11.5	(10.5–12.4)	9.4	(9.0–9.8)
MNAR^a^	6.6	(6.1 ‐ 7.1)	7.3	(6.8–7.7)	11.0	(10.0–12.0)	8.9	(8.5–9.3)
MNAR^b^	7.0	(6.4 ‐ 7.6)	7.8	(7.2–8.3)	11.5	(10.5–12.5)	9.1	(8.7–9.5)
MNAR^c^	7.4	(6.8 ‐ 8.0)	8.3	(7.6–8.9)	11.9	(10.8–13.0)	9.3	(8.9–9.7)
Calibrated	10.2	*14.3*	11.7	*15.6*	16.0	*20.4*	15.5	*20.7*
Calibrated^a^	9.9	*13.8*	11.4	*15.2*	15.2	*19.4*	15.0	*20.2*
Calibrated^b^	9.4	*13.3*	10.9	*14.9*	14.0	*18.0*	14.3	*19.3*
Calibrated^c^	8.9	*12.8*	10.5	*14.7*	13.0	*16.9*	13.7	*18.6*

SD = standard deviation; 95% CI = 95% confidence interval; R = linkage‐consenting Scottish Health Survey respondents (survey‐weighted); MAR = missing‐at‐random; MNAR = missing‐not‐at‐random; ^CR^ = continuum of resistance‐based sensitivity analysis.

a Slight sensitivity analysis;

b moderate sensitivity analysis;

c Extreme sensitivity analyses. Calibrated = calibrated to retail data.

The percentage increase in weekly units consumed from the survey‐weighted estimate to the MAR adjustment among males was typically the greatest in the most deprived quintile (+4.9% in 1995, +3.6% in 1998, +17.8% in 2003 and +13.6% in 2008–2010 compared with −1.6%, +4.4%, −2.6% and −13.6%, respectively, in the least deprived quintile; Table 3; Supporting information, Table S3a–d). Among females, mean consumption was consistently greater in the most deprived quintile both before and after the MAR adjustment and the extent of the adjustment did not follow a pronounced pattern over deprivation (Table 3; Supporting information, Table S3a–d). As the association between harms and consumption is increased progressively through the second MNAR sensitivity analyses (Table 3; Supporting information, Table S3a–d) the gradient over deprivation emerges, in contrast to the survey‐weighted and MAR. These altered gradients are reflected in the corresponding sales data‐calibrated estimates (Table 4; Supporting information, Table S4a–d).

**Table 3:** Weekly alcohol consumption estimates in the 2003 Scottish Health Survey respondents^a^ aged 20–64 years by sex and area deprivation quintile under a range of assumption about the missing data: socio‐demographic based survey weights; MAR; MNAR.

		Survey‐weighted estimates among respondents^a^		MAR estimates in adjusted sample		MNAR^CR^ estimates in adjusted sample		MNAR^b^ estimates in adjusted sample		MNAR^c^ estimates in adjusted sample		MNAR^d^ estimates in adjusted sample	
Quintile of deprivation	*n*	Mean	(95 % CI)	Mean	(95 % CI)	Mean	(95 % CI)	Mean	(95 % CI)	Mean	(95% CI)	Mean	(95 % CI)
Males													
Least deprived	484	23.1	(20.9–25.3)	22.5	(19.3–25.7)	23.9	(20.7–27.1)	23.2	(20.1–26.4)	24.7	(21.2–28.1)	26.1	(22.0–30.2)
2	532	21.4	(19.2–23.6)	20.0	(16.4–23.7)	21.9	(18.2–25.6)	21.4	(17.6–25.1)	24.1	(19.8–28.3)	26.8	(21.6–31.9)
3	500	21.9	(18.8–25.0)	22.8	(18.8–26.9)	24.9	(20.6–29.1)	24.3	(20.0–28.7)	27.3	(22.1–32.5)	30.3	(23.9–36.7)
4	457	20.0	(17.6–22.5)	20.2	(17.4–23.0)	22.9	(20.0–25.9)	22.8	(19.7–25.9)	28.0	(23.6–32.3)	33.1	(27.1–39.2)
Most deprived	380	22.5	(17.7–27.3)	26.5	(18.6–34.4)	31.2	(23.0–39.4)	31.6	(23.3–40.0)	41.9	(32.2–51.6)	52.1	(40.5–63.8)
All quintiles	2353	21.8	(20.5–23.1)	22.4	(20.3–24.4)	24.9	(22.8–27.0)	24.6	(22.4–26.7)	28.9	(26.4–31.5)	33.3	(30.1–36.5)
Females													
Least deprived	603	12.5	(11.4–13.5)	12.9	(11.2–14.5)	13.5	(11.8–15.1)	13.0	(11.3–14.6)	13.2	(11.5–14.8)	13.4	(11.6–15.1)
2	666	12.7	(10.3–15.1)	12.2	(9.4–15.0)	12.8	(10.0–15.6)	12.3	(9.4–15.1)	12.4	(9.6–15.3)	12.6	(9.7–15.5)
3	631	9.7	(8.6–10.8)	9.6	(8.1–11.2)	10.3	(8.8–11.9)	9.8	(8.2–11.4)	10.1	(8.5–11.7)	10.4	(8.7–12.2)
4	586	9.5	(8.3–10.8)	9.4	(7.7–11.1)	10.1	(8.4–11.8)	9.7	(8.0–11.4)	10.2	(8.4–12.1)	10.8	(8.8–12.8)
Most deprived	542	9.4	(7.8–11.0)	9.7	(7.5–11.9)	10.5	(8.3–12.7)	10.2	(8.0–12.5)	11.2	(8.8–13.7)	12.3	(9.6–14.9)
All quintiles	3028	10.8	(10.1–11.6)	10.8	(9.8–11.7)	11.5	(10.5–12.4)	11.0	(10.0–12.0)	11.5	(10.5–12.5)	11.9	(10.8–13.0)

a Scottish Health Survey respondents that have consented to linkage; 95% CI = 95% confidence interval; SD = standard deviation; MAR = missing‐at‐random; MNAR = missing‐not‐at‐random; ^CR^ = continuum of resistance‐based sensitivity analysis.

b Slight sensitivity analysis;

c moderate sensitivity analysis;

d extreme sensitivity analyses. Calibrated = calibrated to retail data.

**Table 4:** Mean weekly alcohol consumption estimates and standard deviations in individuals aged 20 to 64 years in 2003 by sex calibrated to per capita totals.

Quintile of deprivation	Calibrated		Calibrated^CR^		Calibrated^a^		Calibrated^b^		Calibrated^c^	
	Mean	SD	Mean	SD	Mean	SD	Mean	SD	Mean	SD
Males										
Least deprived	33.4	35.6	32.4	30.7	32.1	34.3	30.1	32.6	28.5	31.4
2	29.8	32.1	29.7	29.2	29.6	33.2	29.4	36.8	29.2	40.0
3	33.9	38.4	33.7	37.4	33.7	38.7	33.3	39.8	33.0	40.8
4	30.0	33.4	31.1	31.7	31.6	36.6	34.1	46.0	36.1	54.2
Most deprived	39.4	69.6	42.3	50.4	43.8	72.8	51.1	88.2	56.9	101.7
All quintiles	33.2	41.1	33.7	35.7	34.0	42.3	35.3	47.2	36.4	51.5
Females										
Least deprived	19.1	21.9	18.3	18.5	18.0	20.4	16.1	18.4	14.6	16.8
2	18.0	20.3	17.3	27.4	17.0	19.0	15.2	17.2	13.8	15.7
3	14.3	19.2	14.0	18.7	13.5	18.2	12.3	16.6	11.4	15.5
4	14.0	19.3	13.7	18.2	13.4	18.6	12.5	17.7	11.8	17.0
Most deprived	14.4	19.6	14.2	17.0	14.2	19.2	13.7	18.8	13.4	18.5
All quintiles	16.0	20.4	15.5	20.2	15.2	19.4	14.0	18.0	13.0	16.9
Scaling factor^d^	1.5		1.4		1.4		1.2		1.1	

SD = standard deviation; ^CR^ = continuum of resistance‐based sensitivity analysis.

a Slight sensitivity analysis;

b moderate sensitivity analysis;

c extreme sensitivity analyses. Calibrated = calibrated to retail data.

d Rounded to 1 decimal place.

The unadjusted prevalence of problem drinking was strongly socially patterned—highest in the most deprived quintile—for both sexes (Supporting information, Table S5). MAR adjustment resulted in a proportionally larger change in the more deprived quintiles (Supporting information, Table S5) and had a marginal increase overall (Table 5). The prevalence of binge drinking exhibited a similar social gradient to that of problem drinking, but with a higher prevalence; MAR correction had little impact (Table 4 and Supporting information, Table S6). Non‐drinkers were more prevalent in the most deprived areas; there was negligible effect from MAR correction (Supporting information, Table S7).

**Table 5:** Potential problem‐drinker and binge‐drinking prevalence estimates in the Scottish Health Survey respondents^a^ and in the adjusted sample by survey year, sex and area deprivation quintile.

Survey year(s)	Males				Females			
	Survey‐weighted		MAR		Survey‐weighted		MAR	
	%	(CI)	%	(CI)	%	(CI)	%	(CI)
Potential problem drinking prevalence (among current drinkers)								
1998	12.3	(10.9–13.8)	12.9	(11.5–14.3)	5.0	(4.1–5.8)	5.4	(4.5–6.3)
2003	12.8	(11.2–14.3)	13.3	(11.8–14.7)	6.7	(5.6–7.7)	6.6	(5.6–7.7)
2008/10	14.8	(13.6–16.0)	14.7	(13.6–15.8)	9.1	(8.3–10.0)	8.7	(8.0–9.4)
Binge drinking prevalence (among those who drank in the last 7 days)								
1998	39.7	(37.4–42.0)	39.2	(36.6–41.7)	18.8	(18.6–19.0)	18.6	(16.6–20.7)
2003	34.2	(31.7–36.6)	34.3	(31.5–37.0)	21.1	(19.1–23.2)	20.9	(18.5–23.2)
2008/10	43.2	(41.3–45.0)	43.3	(41.7–44.9)	33.9	(32.3–35.5)	35.7	(34.1–37.2)

a Respondents that have consented to linkage. MAR = missing‐at‐random; 95% CI = 95% confidence interval..

## Discussion

Adjusting for differential survey participation resulted in elevated estimates of weekly alcohol consumption. This was particularly pronounced among men living in the most deprived areas, operating chiefly through the elevated levels of alcohol‐related harm experienced by non‐respondents in this group. Among women, the correction did not have a substantial impact on the level or patterning of weekly consumption. Generally, the prevalence of non‐drinkers and binge drinking were not affected materially by adjustment. For problem drinking, there tended to be a proportionally larger change in the more deprived quintiles. Sensitivity analyses yielded a possible higher range of adjusted estimates of weekly consumption and a steeper social gradient.

Previous studies have shown an association between ill health, including alcohol misuse, and response status 8, 21, 56. However, few studies have used this information to produce adjusted estimates. Recent exceptions considering adjustments to alcohol consumption have assessed the impact of adjustments upon overall consumption level (rather than within subgroups) and found small to non‐existent effects 24, 25. Adjusted estimates for subpopulations of interest are important for evaluation of the impact of policy on inequalities and of heterogeneous or unintended effects 57, 58, and the present study demonstrates that health‐related non‐response may have important differential effects. The most similar study used Swedish registry‐based data on differences in retrospective alcohol‐related hospitalization between survey respondents and inferred estimates for non‐respondents to adjust prevalence estimates of hazardous drinking and abstinence 24. Although those with previous alcohol‐related hospitalizations were, on average, 2.4 times more likely to become survey non‐respondents, adjusting for these differences had little impact upon rates of hazardous alcohol consumption. Potential explanations for differential findings are, first, the proportion hospitalized was low (1.7%), and secondly, the shorter follow‐up period (10 years). In general, our study finds larger impacts of our adjustment, due probably to a longer maximum follow‐up period (16 years from 1995) and higher probabilities of harm (maximum of 6.0% over 16 years in the adjusted sample in 1995) compared with Sweden.

Given declining survey response, the use of auxiliary variables sourced from routine data to make corrections is likely to be of increasing value, particularly as the availability of linked health data increases in many countries 59.

A number of limitations are of note. First, not all survey participants consent to linkage, which may generate distortions, depending on the nature of any differences between non‐consenters and non‐responders. Secondly, the comparison data are not free of bias themselves. Although high—96% in 2001—Census enumeration is incomplete, and under‐enumeration in the 2001 Census was higher among deprived and transient groups 60. Thirdly, the SHeSs’ sampling frame is confined to individuals living in private residences. This excludes a number of marginal population groups: those at high risk of alcohol‐related harm but low access to alcohol (e.g. those incarcerated or in‐treatment), high risk and high access to alcohol groups (e.g. rough sleepers and the armed forces) and those with low risk and low access to alcohol (e.g. long‐term care and nursing homes). Therefore, we would expect to see differences in our comparisons even if the SHeSs represent their target populations accurately. These are likely to be small, as although the excluded group experience higher rates of harm 61 they are small in size 62, 63. Besides, our correction procedure may go some way to generalizing beyond the private‐residing sampling frame. Fourthly, restricting of the analyses to data on individuals aged 20–64 years makes comparison with sales data more challenging, as they consume more than older and younger groups per capita. This was taken account of by increasing the proportional difference in mean consumption, as detailed in Supporting information, Appendix S3. Fifthly, more information was available to inform corrections in the earlier survey years due to longer follow‐up. Therefore, adjusted time trends should be interpreted with caution, as the magnitude of the adjustment in any year is a function of this differential level of information available to inform corrections, in addition to any real differences in level and impact of non‐response over time. There have also been refinements to the ways in which data on alcohol consumption are collected, so pooling and direct comparison between the pre‐ and post‐2003 data are not recommended 29. We are thus unable to determine the extent to which changes in consumption estimates are attributable to the changes in response levels over time. Sixthly, the use of information on alcohol‐related harms in our methodology was motivated specifically by the objective of refining alcohol consumption estimates and the extent to which it is informative for other health behaviours is limited. Finally, unlike countries operating national registries, with unique individual identification and comprehensive linkage 64, attributes of individual non‐respondents cannot be identified explicitly from our linked data and have to be inferred based on reference to general population data. Validation of this approach is a potential future avenue of research.

The results yielded some initially unexpected findings. First, adjustment had little impact upon binge drinking estimates. This could be because binge drinking relates to questions concerning drinking in the last 7 days, whereas the consumption estimates relate to usual drinking in the previous year and problem drinking relates to ever occurrence. As such, the binge drinking measure itself is likely to be less stable and, on investigation, there was weaker association between harms and binge drinking (than, e.g. problem drinkers). Another factor could be a weak association between missingness and binge drinking, but this was untestable with the available data. Second, in some cases (e.g. for women), the sales data‐calibrated figures decreased within subgroups as the MNAR assumptions become more extreme. This was found to result from the decreasing scaling factors of the calibration process: for subgroups for which the non‐response estimates do not change greatly going from MAR to MNAR***, the decreasing scaling factors ‘outweigh’ the small increases in non‐response adjusted estimates.

This study exploited record‐linked survey data and comparison population data to identify health‐related deviations from representativeness among survey respondents aged 20–64 years in Scotland. Identified differences were then used to adjust key measures of alcohol consumption in an innovative way. The findings indicated that alcohol‐related non‐representativeness may have little impact on estimates of women's alcohol consumption but, for men, overall levels and socio‐economic gradients in consumption were underestimated. The study provides a guide to the magnitude of the effect of the universal limitation of non‐response in alcohol studies and illustrates new methods for triangulation of alcohol consumption estimates. The methodology has utility for refinement of measures of other health‐related behaviours such as smoking with corresponding informative outcomes (smoking‐related deaths and hospitalization for smoking‐related causes) wherever survey data have been record linked to relevant data.

### Declaration of interests

G.McC., L.G. and M.R. were members of the Scottish Government‐funded MESAS (Monitoring and Evaluating Scotland's Alcohol Strategy) evaluation. The remaining authors declare that they have no competing interests.

## Supporting information


**Appendix S1** Specification of missing at random (MAR) imputation models.
**Appendix S2** Specification of missing not at random (MNAR) imputation models.
**Appendix S3** Calibration to retail data totals.
**Table S1** Sex‐ and area deprivation quintile‐specific breakdowns for the general population of Scotland and respondents to the Scottish Health Survey 1995 to 2008–2010 with inferred estimates for non‐respondents.
**Table S2** The probabilities of alcohol‐related harm in the population, in the Scottish Health Survey respondents and the synthetic non‐respondents by survey year, sex and area deprivation quintile during follow‐up period.
**Table S3** Problem‐drinking prevalence estimates in the Scottish Health Survey respondents, and adjusted estimates under missing‐at‐random by survey year, sex and area deprivation quintile.
**Table S4** Binge‐drinking prevalence estimates in the Scottish Health Survey respondents, and adjusted estimates under missing‐at‐random by survey year, sex and area deprivation quintile.
**Table S5** Non‐drinker prevalence estimates in the Scottish Health Survey respondents, and adjusted estimates under missing‐at‐random by survey year, sex and area deprivation quintile.
**Table S6** Weekly alcohol consumption estimates in the Scottish Health Survey respondents aged 20–64 years by sex and area deprivation quintile under a range of assumption about the missing data: socio‐demographic based survey weights; missing at random (MAR); missing not at random (MNAR).
**Table S7** Weekly alcohol consumption estimates in the Scottish Health Survey respondents aged 20–64 years by sex and area deprivation quintile calibrated to per capita estimates.
