# Supplementary material for: Adjustment for survey non‐representativeness using record‐linkage: refined estimates of alcohol consumption by deprivation in Scotland
Source: Addiction. 2017 Apr 25;112(7):1270–80. doi: 10.1111/add.13797 (PMC5467727; doi:10.1111/add.13797)
Supplement: Supplementary file 1 — Appendix S1 Specification of missing at random (MAR) imputation models. Appendix S2 Specification of missing not at random (MNAR) imputation models. Appendix S3 Calibration to retail data totals. Table S1 Sex‐ and area deprivation quintile‐specific breakdowns for the general population of Scotland and respondents to the Scottish Health Survey 1995 to 2008–2010 with inferred estimates for non‐respondents. Table S2 The probabilities of alcohol‐related harm in the population, in the Scottish Health Survey respondents and the synthetic non‐respondents by survey year, sex and area deprivation quintile during follow‐up period. Table S3 Problem‐drinking prevalence estimates in the Scottish Health Survey respondents, and adjusted estimates under missing‐at‐random by survey year, sex and area deprivation quintile. Table S4 Binge‐drinking prevalence estimates in the Scottish Health Survey respondents, and adjusted estimates under missing‐at‐random by survey year, sex and area deprivation quintile. Table S5 Non‐drinker prevalence estimates in the Scottish Health Survey respondents, and adjusted estimates under missing‐at‐random by survey year, sex and area deprivation quintile. Table S6 Weekly alcohol consumption estimates in the Scottish Health Survey respondents aged 20–64 years by sex and area deprivation quintile under a range of assumption about the missing data: socio‐demographic based survey weights; missing at random (MAR); missing not at random (MNAR). Table S7 Weekly alcohol consumption estimates in the Scottish Health Survey respondents aged 20–64 years by sex and area deprivation quintile calibrated to per capita estimates. [file ADD-112-1270-s001.docx]

**Adjustment for survey non-representativeness using record-linkage: A proof of principle study to develop refined estimates of alcohol consumption in Scotland**

## Web Materials

### Contents

Supplementary Appendix S1: Specification of MAR imputation models

Supplementary Appendix S2: Specification of MNAR imputation models

Supplementary Appendix S3: Calibration to retail data totals

Supplementary Table S1: Sex- and area deprivation quintile-specific breakdowns for the general population of Scotland and respondents to the Scottish Health Survey 1995 to 2008-2010 with inferred estimates for non-respondents.

Supplementary Table S2: The probabilities of alcohol-related harm in the population, in the Scottish Health Survey respondents and the synthetic non-respondents by survey year, sex and area deprivation quintile during follow-up period

### Supplementary Table S3: Problem- drinking prevalence estimates in the Scottish Health Survey respondents, and adjusted estimates under missing-at-random by survey year, sex and area deprivation quintile

Supplementary Table S4: Binge-drinking prevalence estimates in the Scottish Health Survey respondents, and adjusted estimates under missing-at-random by survey year, sex and area deprivation quintile

Supplementary Table S5: Non-drinker prevalence estimates in the Scottish Health Survey respondents, and adjusted estimates under missing-at-random by survey year, sex and area deprivation quintile

Supplementary Table S6: Weekly alcohol consumption estimates in the Scottish Health Survey respondents aged 20-to-64-years by sex and area deprivation quintile under a range of assumption about the missing data: socio-demographic based survey weights; MAR; MNAR

Supplementary Table S7: Weekly alcohol consumption estimates in the SHeS respondents aged 20-to-64-years by sex and area deprivation quintile calibrated to per capita estimates

### Supplementary Appendix S1: Specification of missing-at-random (MAR) imputation model

Under MAR, the joint distribution of the outcome given the observed covariates is assumed equivalent for respondents and non-respondents. Measures imputed were: usual weekly alcohol consumption measured using the Quantity-Frequency method; the prevalence of non-drinkers; alcohol consumption on the heaviest drinking day in the last seven days and potential problem-drinking—defined as two or more positive answers on the CAGE instrument.

Usual weekly alcohol consumption includes both those who have zero consumption, and a continuous varying component of positive consumption. To capture these features a two-stage process was employed: first modeling the binary outcome of current drinking status, and then the level of consumption was modeled conditional on being a current drinker. A small number of respondents (*n*=0, 19, 16, 12 in 1995, 1998, 2003 and 2008/10 respectively) had missing alcohol consumption which was imputed in the same way as for non-respondents. Weekly consumption was set to zero for those who are imputed as non-drinkers. Positive consumption was transformed to approximate Normality using the shifted-log transformation$f\left( y \right)=\ln(\pm y-a)$, where the sign of $y$ and the value of $a$ are estimated such that $f\left( y \right)$ is positive and has near zero skewness (1). This was implemented using the *-lnskew0*- function in Stata. A similar approach was taken to impute the amount consumed on the heaviest drinking day of the previous week. First, whether an individual drunk alcohol during the previous week was imputed using a logit model; then the amount consumed was then imputed conditional on drinking alcohol in the previous week. Problem drinking was imputed using a logit imputation model. Models were fitted separately by sex- and deprivation- subgroup. These models were specified jointly in -ice- (2) to produce 70 imputed datasets. Predictive mean matching with a potential match pool of size 10 was used to improve the imputations. All imputations models included the standard survey weights to account for survey design. Survey weighted estimates of mean weekly consumption, prevalence of binge drinking, problem drinking and abstinence were derived for each dataset and combined using Rubin’s rules (3).

**Supplementary Appendix S2:** Specification of MNAR imputation models

This section describes the specification of the MNAR imputation models(4). MNAR allows the distribution of the outcome given the observed covariates to differ between non-respondents and respondents. The difference between these conditional distributions is determined by a sensitivity parameter $\delta$: the adjusted mean difference in the outcome between respondents and non-respondents. This difference cannot be identified from our data, without making untestable distributional assumptions or parameter restrictions. We proceed by specifying this value based on plausible hypotheses about differences between respondents and non-respondents, with reference to external data.

In this way, we modified the imputation procedure for the outcome mean weekly alcohol consumption. The first stage of the MAR imputation model, for the binary outcome of drinking status, was left as the under MAR. For the second stage, we consider a MNAR specification which allows differential alterations of the imputation model by harms ($H_{i}$) and by sex ${(S}_{i}).$

$Y_{i}^{*}|Z_{i}=1,R_{i}\sim N\left( \alpha_{Y}+\beta_{Y}\boldsymbol{X}_{\boldsymbol{i}}+\gamma_{Y}\boldsymbol{H}_{\boldsymbol{i}}+\zeta_{Y}w_{i}+ \left( 1-R_{i} \right)\left( \delta_{0}^{S_{i}}\left( 1-\boldsymbol{H}_{\boldsymbol{i}} \right)+\delta_{1}^{S_{i}}\boldsymbol{H}_{\boldsymbol{i}} \right),\sigma^{2} \right).$

Here $w_{i}$ denotes the standard survey weights; $Z_{i}$ indicates current drinking status; $R_{i}$indicates response status. Compared to respondents with fully observed alcohol consumption, mean alcohol consumption is modified by $\delta_{1}^{S_{i}}$ among the non-respondents who experienced harms, depending on sex ${(S}_{i})$; and similarly by $\delta_{0}^{S_{i}}$ among non-respondents who do not experience harms. MAR is the case $\delta_{0}^{S_{i}}=\delta_{1}^{S_{i}}=0$.

**Supplementary Appendix S3**: Calibration to retail data totals

Alcohol sales data can be used to provide estimates of per capita consumption closest to a “gold standard” for alcohol measurement (5), although evidence is accruing that this remains an underestimation of true national consumption (6). It is, however, limited by its aggregate nature, and it is useful to combine multiple sources of data to “triangulate” estimates of sub-group alcohol consumption (5). This has been performed previously by Rehm *et al* (7) using US data, and more recently Meier *et al* (8) for England and Wales. To calibrate our non-response adjusted survey estimates to the aggregate retail figures, we follow previous approaches which have favored the use of a Gamma distribution to model the population distribution of positive alcohol consumption (7). In this paper we are interested in average population alcohol exposure—that is, mean alcohol consumption including non-drinkers (9). For this calibration exercise, we model average alcohol consumption and its standard deviation using a Bernoulli-Gamma mixture distribution. The choice to drink is modeled as a Bernoulli random variable, and the level of consumption among drinkers is modeled using a two-parameter Gamma distribution.

To calibrate non-response adjusted survey estimated to the per-capita retail data estimates, drawing on the scaling property of the Gamma distribution, the survey distribution of positive alcohol consumption was scaled upward by a constant proportional factor denoted *c*. In other words, a *constant* *rate* of under-reporting across is assumed. In reality, under-reporting is likely to vary between sub-groups and by amount consumed within sub-groups, but in the absence of concrete information to guide the specification of differential under-reporting, constant under-reporting represents a pragmatic approach.

This scaling factor was computed as the proportionate difference in mean consumption between the non-response adjusted estimate of population mean consumption and the per capita retail figure. However, as the survey correction was computed for a restricted age-range (20-to-64), computing this scaling factor by comparing the restricted age-range mean and the 16+ per-capita figure would effectively assume constant per-capita consumption over all ages. Best evidence (weighted survey estimates among the full sample including non-consenters to linkage) indicates older and younger groups consume less on average than those aged 20-to-64. To take this into account, the 16+ per-capita figure was increased by the proportional difference in mean consumption between 16+ and working age from the survey. The scaling factor was computed as the proportion difference between this age-group adjusted retail figure and the MNAR estimate.

Weekly alcohol consumption $y_{i}$ can be characterised as a mixture of a Bernoulli random variable, representing the decision to drink and a Gamma random variable characterising the distribution of positive values of alcohol consumption. Let *i* index survey and synthetic records *i=*1*…N*. Suppose the positive component follows Gamma distribution with parameters (*k,*$\theta$), where *k* is a shape parameter and $\theta$ is a scale parameter. The Gamma p.d.f. can be written as follows:

$$f\left( y_{i} \right)=\frac{1}{\Gamma(k)\theta^{k}}y_{i}^{k-1}e^{\frac{-y_{i}}{\theta}}$$

$0<y_{i}<\infty$; $0<\theta$ ; $0<k$

Parameters (*k,*$\theta$) can be estimated using maximum-likelihood. The mean and variance can also be expressed in terms of these parameters.

$$\mathbb{E}\left( y_{i} \right)=k\theta$$

$$V\left( y_{i} \right)=k\theta^{2}$$

The Gamma distribution has the scaling property (10): multiplying a Gamma random variable $y_{i}$ by a positive constant *c* results in another Gamma distributed variable with parameters (*k,*$c\theta$). Characterizing positive population consumption as a Gamma random variable, $y_{P,i} ,$scaled upward from the survey data by a positive constant *c,* then the population distribution can be defined as follows:

$y_{P,i}=cy_{i}\sim Gamma (k,c\theta$*)*

$$\mathbb{E}\left( y_{P,i} \right)=ck\theta=c\mathbb{E}\left( y_{i} \right)$$

$$V\left( y_{P,i} \right)=k({c\theta)}^{2}$$

Given $c$, $k$ and $\theta$are known, this gives sufficient information to compute the mean and variance of a scaled-up distribution of positive weekly alcohol consumption as a simple transformation of the survey distribution, based on a constant scaling factor of *c*.

In this paper we examine the full distribution of consumption—including zero values A mixture of the Gamma distribution and a distribution characterising the decision to drink or abstain extends the above to include zero values.

The Bernoulli-Gamma distribution is described as follows:$f\left( y_{i} | \theta,k,\pi\right)=\left\{ \begin{aligned} 1-\pi\text{if }y_{i}=0 \\ \pi\frac{1}{\Gamma(k)\theta^{k}}y_{i}^{k-1}e^{\frac{-y_{i}}{\theta}}\text{ if }y_{i}>0 \end{aligned} \right.$

Where $\pi$ is the probability being a drinker,*k* is the shape parameter and $\theta$ is the scale parameter.

The mean and variance of this distribution is:

$$\mathbb{E}\left( y_{i} \right)=\pi k\theta$$

$$V\left( y_{i} \right)=\pi(1-\pi){(k\theta)}^{2}+\pi k\theta^{2}$$

Substituting the parameters of the scaled-up Gamma distribution, where$\theta_{P}$=$c\theta$

$\mathbb{E}\left( y_{P,i} \right)=\pi k\theta_{P}$*=*$\pi\mathbb{E}\left( y_{i} \right)c$

$$V\left( y_{P,i} \right)=\pi(1-\pi){(k\theta_{P})}^{2}+\pi k{\theta_{P}}^{2}$$

Therefore the mean and variance of the scaled-up distribution can be computed using the fitted shape and scale parameters ($\theta,k)$; the MAR-adjusted sample proportion of drinkers ($\pi$); and the scaling constant (c). We also derived the scaled-up mean and standard deviations separately within sex, and sex-deprivation-quintile, sub-groups. We allowed the parameters of the distribution of positive consumption, $\theta,k,$ and the non-response adjusted proportion of drinkers, $\pi,$ to vary by these subgroups. In contrast, *c* was not varied between subgroups. In reality, *c is* likely to vary within and between sub-groups, however constant *c* is a pragmatic starting point. Recent evidence from Canada (11) found no gender differences in under-reporting of alcohol in a telephone survey, which may provide support for *c* being constant over gender. An interesting finding was that low-risk drinkers appeared to under-report more than high-risk drinkers. This would be an interesting feature to incorporate in further work.

# Supplementary Table S1: Sex- and area deprivation quintile-specific breakdowns (%) for the general population of Scotland and respondents† to the Scottish Health Survey 1995 to 2008-2010 aged 20-to-64-years with inferred estimates for non-respondents and the combined “adjusted sample”.

|  | Population | | SHeS | | | | | |
| --- | --- | --- | --- | --- | --- | --- | --- | --- |
| Quintile of deprivation |  | | Respondents* | | Inferred non-respondents | | Inferred total | |
|  | Males (%) | Females (%) | Males (%) | Females (%) | Males (%) | Females (%) | Males (%) | Females (%) |
|  |  |  |  |  |  |  |  |  |
|  |  |  |  | *1995* |  |  |  |  |
|  |  |  |  |  |  |  |  |  |
| Least deprived | 11.3 | 11.4 | 9.7 | 10.1 | 12.4 | 12.3 | 11.3 | 11.4 |
| 2 | 10.0 | 10.3 | 9.8 | 10.0 | 10.2 | 10.5 | 10.0 | 10.3 |
| 3 | 9.7 | 10.1 | 10.6 | 9.5 | 9.2 | 10.4 | 9.7 | 10.1 |
| 4 | 9.4 | 9.9 | 9.4 | 8.8 | 9.4 | 10.7 | 9.4 | 9.9 |
| Most deprived | 8.6 | 9.3 | 10.4 | 11.8 | 7.3 | 7.6 | 8.6 | 9.3 |
| *All quintiles* | 49.0 | 51.0 | 49.8 | 50.2 | 48.5 | 51.5 | 49.0 | 51.0 |
|  |  |  |  |  |  |  |  |  |
|  |  |  |  | *1998* |  |  |  |  |
|  |  |  |  |  |  |  |  |  |
| Least deprived | 11.4 | 11.6 | 10.4 | 10.2 | 12.1 | 12.5 | 11.4 | 11.6 |
| 2 | 10.1 | 10.3 | 9.5 | 8.7 | 10.5 | 11.5 | 10.1 | 10.3 |
| 3 | 9.7 | 10.1 | 10.7 | 11.1 | 9.1 | 9.4 | 9.7 | 10.1 |
| 4 | 9.4 | 9.9 | 9.4 | 10.2 | 9.3 | 9.7 | 9.4 | 9.9 |
| Most deprived | 8.4 | 9.1 | 9.9 | 9.9 | 7.3 | 8.6 | 8.4 | 9.1 |
| *All quintiles* | 48.9 | 51.1 | 50.0 | 50.0 | 48.2 | 51.8 | 48.9 | 51.1 |
|  |  |  |  |  |  |  |  |  |
|  |  |  |  | *2003* |  |  |  |  |
|  |  |  |  |  |  |  |  |  |
| Least deprived | 10.4 | 10.5 | 10.5 | 11.4 | 10.1 | 9.3 | 10.4 | 10.5 |
| 2 | 10.0 | 10.2 | 10.2 | 10.9 | 9.8 | 9.3 | 10.0 | 10.2 |
| 3 | 9.7 | 10.0 | 9.1 | 9.7 | 10.6 | 10.5 | 9.7 | 10.0 |
| 4 | 9.6 | 10.2 | 9.7 | 10.3 | 9.5 | 10.0 | 9.6 | 10.2 |
| Most deprived | 9.1 | 10.1 | 8.4 | 9.9 | 10.3 | 10.5 | 9.1 | 10.1 |
| *All quintiles* | 48.9 | 51.1 | 47.9 | 52.1 | 50.3 | 49.7 | 48.9 | 51.1 |
|  |  |  |  |  |  |  |  |  |
|  |  |  | *2008-10* | | |  |  |  |
|  |  |  |  | | |  |  |  |
| Least deprived | 10.3 | 10.5 | 9.4 | 9.9 | 11.7 | 11.5 | 10.3 | 10.5 |
| 2 | 10.3 | 10.5 | 9.9 | 10.2 | 11.0 | 11.1 | 10.2 | 10.3 |
| 3 | 10.0 | 10.2 | 9.3 | 9.6 | 11.3 | 11.4 | 10.0 | 10.2 |
| 4 | 9.5 | 9.9 | 9.8 | 10.7 | 8.9 | 8.6 | 9.5 | 9.9 |
| Most deprived | 8.9 | 9.8 | 9.8 | 11.3 | 7.4 | 7.2 | 9.1 | 9.9 |
| *All quintiles* | 49.0 | 51.0 | 48.3 | 51.7 | 50.3 | 49.7 | 49.1 | 50.9 |

† Respondents that have consented to linkage

**Supplementary Table S2**: The probabilities of alcohol-related harm in the population, in the Scottish Health Survey respondents* and the synthetic non-respondents and the combined “adjusted sample|” for those aged 20-to-64-years by survey year, sex and area deprivation quintile during follow-up period.

|  | Population | | SHeS | | | | | |
| --- | --- | --- | --- | --- | --- | --- | --- | --- |
| Quintile of deprivation |  | | Respondents† | | Inferred non-respondents | | Inferred total | |
|  | Males (%) | Females (%) | Males (%) | Females (%) | Males (%) | Females (%) | Males (%) | Females (%) |
|  |  |  |  |  |  |  |  |  |
|  |  |  |  | *1995* |  |  |  |  |
|  |  |  |  |  |  |  |  |  |
| Least deprived | 3.4 | 1.7 | 4.1 | 1.5 | 3 | 2 | 3.5 | 1.7 |
| 2 | 5.9 | 2.7 | 4.0 | 1.7 | 10 | 5 | 5.6 | 2.6 |
| 3 | 7.9 | 3.4 | 4.9 | 3.0 | 20 | 4 | 7.3 | 3.4 |
| 4 | 10.4 | 4.3 | 6.6 | 2.8 | 29 | 8 | 11.1 | 4.3 |
| Most deprived | 17.2 | 6.3 | 9.5 | 3.4 | 58 | 49 | 15.9 | 6.2 |
| *All quintiles* | 8.9 | 3.7 | 5.9 | 2.5 | 16.7 | 6.6 | 8.4 | 3.6 |
|  |  |  |  |  |  |  |  |  |
|  |  |  |  | *1998* |  |  |  |  |
|  |  |  |  |  |  |  |  |  |
| Least deprived | 2.9 | 1.4 | 2.4 | 1.1 | 4.0 | 2.1 | 2.8 | 1.4 |
| 2 | 5.1 | 2.3 | 3.3 | 2.2 | 7.9 | 2.8 | 4.6 | 2.4 |
| 3 | 6.9 | 3.0 | 3.8 | 1.1 | 16.7 | 7.7 | 6.2 | 2.3 |
| 4 | 9.2 | 3.8 | 4.7 | 2.8 | 18.9 | 6.9 | 7.9 | 3.8 |
| Most deprived | 15.3 | 5.6 | 11.5 | 3.7 | 31.4 | 10.4 | 14.3 | 5.0 |
| *All quintiles* | 7.9 | 3.2 | 5.1 | 2.2 | 12.8 | 5.1 | 6.9 | 2.9 |
|  |  |  |  |  |  |  |  |  |
|  |  |  |  | *2003* |  |  |  |  |
|  |  |  |  |  |  |  |  |  |
| Least deprived | 1.9 | 0.9 | 1.2 | 0.7 | 2.7 | 1.3 | 1.9 | 0.9 |
| 2 | 3.0 | 1.4 | 1.3 | 1.6 | 4.9 | 1.2 | 2.9 | 1.5 |
| 3 | 4.3 | 2.0 | 3.6 | 2.1 | 5.0 | 1.8 | 4.2 | 1.9 |
| 4 | 6.6 | 2.8 | 4.2 | 2.2 | 9.3 | 3.4 | 6.5 | 2.8 |
| Most deprived | 11.4 | 4.2 | 5.8 | 2.6 | 16.4 | 5.9 | 11.1 | 4.2 |
| *All quintiles* | 5.4 | 2.3 | 3.1 | 1.8 | 7.6 | 2.8 | 5.2 | 2.2 |
|  |  |  |  |  |  |  |  |  |
|  |  |  | *2008-10* | | |  |  |  |
|  |  |  |  | | |  |  |  |
| Least deprived | 0.5 | 0.3 | 0.2 | 0.4 | 0.0 | 0.0 | 0.1 | 0.2 |
| 2 | 0.8 | 0.4 | 0.6 | 0.3 | 0.1 | 0.0 | 0.4 | 0.2 |
| 3 | 1.3 | 0.6 | 0.8 | 0.2 | 1.0 | 0.2 | 0.9 | 0.2 |
| 4 | 2.2 | 0.9 | 1.1 | 0.7 | 3.7 | 1.6 | 2.3 | 1.1 |
| Most deprived | 4.2 | 1.5 | 2.4 | 1.3 | 9.0 | 2.9 | 5.1 | 2.0 |
| *All quintiles* | 1.8 | 0.7 | 1.0 | 0.6 | 2.4 | 0.9 | 1.7 | 0.7 |

† Respondents that have consented to linkage

**Supplementary Table S3a**: Weekly alcohol consumption estimates in the 1995 Scottish Health Survey respondents† aged 20-to-64-years by sex and area deprivation quintile under a range of assumption about the missing data: socio-demographic based survey weights; MAR; MNAR.

|  |  | Survey-weighted estimates among respondents† | | | | MAR estimates in adjusted sample | | | | MNAR^CR^ estimates in adjusted sample | | | | MNAR^*^ estimates in adjusted sample | | | | MNAR^**^ estimates in adjusted sample | | | | MNAR^***^ estimates in adjusted sample | | | |
| --- | --- | --- | --- | --- | --- | --- | --- | --- | --- | --- | --- | --- | --- | --- | --- | --- | --- | --- | --- | --- | --- | --- | --- | --- | --- |
|  |  |  |  |  |  |  |  |  |  |  |  |  |  |  |  |  |  |  |  |  |  |  |  |  |  |
| Quintile of deprivation | N | Mean | (95% CI) | | Mean | | (95% CI) | | Mean | | (95% CI) | | Mean | | (95% CI) | | Mean | | (95% CI) | | Mean | | (95% CI) | |  |
| *Males* |  |  |  | |  | |  | |  | |  | |  | |  | |  | |  | |  | |  | |  |
| Least deprived | 566 | 19.2 | (17.3 - 21.1) | | 18.9 | | (16.9 - 20.8) | | 19.8 | | (17.8 - 21.8) | | 19.2 | | (17.2 - 21.3) | | 20.0 | | (17.5 - 22.4) | | 20.7 | | (17.6 - 23.7) | |  |
| 2 | 637 | 19.4 | (17.3 - 21.4) | | 19.8 | | (17.5 - 22.1) | | 21.0 | | (18.6 - 23.4) | | 20.9 | | (18.4 - 23.4) | | 23.0 | | (19.8 - 26.3) | | 25.2 | | (20.9 - 29.4) | |  |
| 3 | 675 | 18.8 | (16.9 - 20.7) | | 19.0 | | (17.0 - 21.0) | | 20.2 | | (18.0 - 22.4) | | 20.4 | | (18.1 - 22.7) | | 23.1 | | (19.7 - 26.5) | | 25.8 | | (21.0 - 30.6) | |  |
| 4 | 572 | 20.0 | (18.0 - 22.0) | | 21.4 | | (18.2 - 24.5) | | 23.2 | | (20.0 - 26.4) | | 23.7 | | (20.4 - 27.0) | | 28.4 | | (24.2 - 32.7) | | 33.1 | | (27.4 - 38.8) | |  |
| Most deprived | 668 | 26.5 | (22.6 - 30.4) | | 27.8 | | (23.9 - 31.7) | | 30.0 | | (25.9 - 34.0) | | 30.9 | | (26.8 - 35.1) | | 37.1 | | (31.9 - 42.4) | | 43.4 | | (36.5 - 50.2) | |  |
| *All quintiles* | 3,118 | 20.8 | (19.7 - 22.0) | | 21.2 | | (20.0 - 22.4) | | 22.6 | | (21.4 - 23.8) | | 22.8 | | (21.5 - 24.1) | | 25.9 | | (24.2 - 27.7) | | 29.1 | | (26.7 - 31.4) | |  |
|  |  |  |  | |  | |  | |  | |  | |  | |  | |  | |  | |  | |  | |  |
| *Females* |  |  |  | |  | |  | |  | |  | |  | |  | |  | |  | |  | |  | |  |
| Least deprived | 690 | 7.2 | (6.5 - 8.0) | | 7.5 | | (6.6 - 8.5) | | 7.8 | | (6.9 - 8.8) | | 7.6 | | (6.6 - 8.6) | | 7.8 | | (6.8 - 8.8) | | 7.9 | | (6.8 - 9.0) | |  |
| 2 | 820 | 5.7 | (5.2 - 6.3) | | 5.9 | | (5.2 - 6.6) | | 6.1 | | (5.4 - 6.9) | | 6.0 | | (5.3 - 6.8) | | 6.4 | | (5.6 - 7.1) | | 6.7 | | (5.8 - 7.6) | |  |
| 3 | 770 | 5.4 | (4.9 - 5.8) | | 5.4 | | (4.8 - 6.1) | | 5.7 | | (5.1 - 6.3) | | 5.6 | | (4.9 - 6.2) | | 5.9 | | (5.1 - 6.6) | | 6.2 | | (5.3 - 7.1) | |  |
| 4 | 680 | 6.7 | (4.8 - 8.6) | | 6.7 | | (4.7 - 8.7) | | 7.0 | | (4.9 - 9.0) | | 7.0 | | (4.9 - 9.0) | | 7.5 | | (5.4 - 9.6) | | 8.1 | | (5.9 - 10.2) | |  |
| Most deprived | 907 | 6.2 | (5.6 - 6.9) | | 6.4 | | (5.7 - 7.2) | | 6.5 | | (5.8 - 7.3) | | 6.8 | | (6.0 - 7.6) | | 7.5 | | (6.5 - 8.5) | | 8.2 | | (6.9 - 9.4) | |  |
| *All quintiles* | 3,867 | 6.3 | (5.8 - 6.7) | | 6.4 | | (5.9 - 6.9) | | 6.7 | | (6.2 - 7.2) | | 6.6 | | (6.1 - 7.1) | | 7.0 | | (6.4 - 7.6) | | 7.4 | | (6.8 - 8.0) | |  |
|  |  |  | |  | |  | |  | |  | |  | |  | |  | |  | |  | |  | |  | |

†: respondents that have consented to linkage; 95% CI: 95% confidence interval; SD: standard deviation; MAR: missing-at-random; MNAR: missing-not-at-random; ^CR^ continuum of resistance-based sensitivity analysis; ^*^ slight sensitivity analysis; ^**^ moderate sensitivity analysis; ^***^ extreme sensitivity analyses.

**Supplementary Table S3b**: Weekly alcohol consumption estimates in the 1998 Scottish Health Survey respondents† aged 20 to 64 years by sex and area deprivation quintile under a range of assumption about the missing data: socio-demographic based survey weights; MAR; MNAR.

|  |  | Survey-weighted estimates among respondents* | | | | MAR estimates in adjusted sample | | | | MNAR^CR^ estimates in adjusted sample | | | | MNAR^*^ estimates in adjusted sample | | | | MNAR^**^ estimates in adjusted sample | | | | MNAR^***^ estimates in adjusted sample | | | |
| --- | --- | --- | --- | --- | --- | --- | --- | --- | --- | --- | --- | --- | --- | --- | --- | --- | --- | --- | --- | --- | --- | --- | --- | --- | --- |
|  |  |  |  |  |  |  |  |  |  |  |  |  |  |  |  |  |  |  |  |  |  |  |  |  |  |
| Quintile of deprivation | N | Mean | (95% CI) | | Mean | | (95% CI) | | Mean | | (95% CI) | | Mean | | (95% CI) | | Mean | | (95% CI) | | Mean | | (95% CI) | |  |
| *Males* |  |  |  | |  | |  | |  | |  | |  | |  | |  | |  | |  | |  | |  |
| Least deprived | 541 | 18.3 | (16.6 - 20.1) | | 19.1 | | (16.5 - 21.8) | | 20.1 | | (17.5 - 22.8) | | 19.7 | | (17.1 - 22.4) | | 20.8 | | (18.0 - 23.7) | | 22.0 | | (18.6 - 25.3) | |  |
| 2 | 615 | 19.1 | (17.1 - 21.0) | | 20.0 | | (17.0 - 23.0) | | 21.3 | | (18.2 - 24.3) | | 21.1 | | (18.0 - 24.1) | | 23.3 | | (19.7 - 26.8) | | 25.4 | | (21.1 - 29.8) | |  |
| 3 | 680 | 18.1 | (16.5 - 19.8) | | 18.6 | | (16.4 - 20.8) | | 20.0 | | (17.8 - 22.3) | | 20.2 | | (17.9 - 22.5) | | 23.4 | | (20.4 - 26.5) | | 26.6 | | (22.5 - 30.7) | |  |
| 4 | 531 | 19.6 | (17.5 - 21.6) | | 21.2 | | (17.9 - 24.5) | | 23.1 | | (19.7 - 26.5) | | 23.4 | | (19.9 - 26.9) | | 27.9 | | (23.5 - 32.2) | | 32.3 | | (26.8 - 37.9) | |  |
| Most deprived | 577 | 25.0 | (21.9 - 28.2) | | 25.9 | | (22.5 - 29.3) | | 27.7 | | (24.1 - 31.3) | | 28.2 | | (24.5 - 32.0) | | 32.9 | | (27.7 - 38.0) | | 37.5 | | (30.6 - 44.4) | |  |
| *All quintiles* | 2,944 | 20.0 | (19.0 - 21.0) | | 20.8 | | (19.5 - 22.0) | | 22.2 | | (20.9 - 23.6) | | 22.3 | | (20.9 - 23.6) | | 25.3 | | (23.5 - 27.0) | | 28.2 | | (26.0 - 30.5) | |  |
|  |  |  |  | |  | |  | |  | |  | |  | |  | |  | |  | |  | |  | |  |
| *Females* |  |  |  | |  | |  | |  | |  | |  | |  | |  | |  | |  | |  | |  |
| Least deprived | 655 | 7.6 | (6.8 - 8.3) | | 7.1 | | (6.3 - 8.0) | | 7.5 | | (6.7 - 8.3) | | 7.3 | | (6.4 - 8.1) | | 7.5 | | (6.6 - 8.4) | | 7.8 | | (6.7 - 8.8) | |  |
| 2 | 685 | 7.3 | (6.6 - 8.0) | | 7.4 | | (6.5 - 8.4) | | 7.8 | | (6.9 - 8.8) | | 7.6 | | (6.6 - 8.6) | | 7.9 | | (6.9 - 9.0) | | 8.3 | | (7.0 - 9.6) | |  |
| 3 | 892 | 6.7 | (6.0 - 7.4) | | 7.0 | | (5.9 - 8.1) | | 7.2 | | (6.1 - 8.3) | | 7.3 | | (6.1 - 8.4) | | 7.8 | | (6.6 - 9.0) | | 8.4 | | (7.1 - 9.8) | |  |
| 4 | 724 | 6.4 | (5.7 - 7.2) | | 6.5 | | (5.4 - 7.6) | | 6.7 | | (5.6 - 7.9) | | 6.8 | | (5.6 - 7.9) | | 7.4 | | (6.1 - 8.6) | | 8.0 | | (6.6 - 9.4) | |  |
| Most deprived | 718 | 7.0 | (6.0 - 8.0) | | 7.0 | | (6.0 - 8.1) | | 7.3 | | (6.2 - 8.4) | | 7.4 | | (6.4 - 8.5) | | 8.2 | | (6.9 - 9.5) | | 9.0 | | (7.4 - 10.7) | |  |
| *All quintiles* | 3,674 | 7.0 | (6.6 - 7.3) | | 7.0 | | (6.5 - 7.5) | | 7.3 | | (6.9 - 7.8) | | 7.3 | | (6.8 - 7.7) | | 7.8 | | (7.2 - 8.3) | | 8.3 | | (7.6 - 8.9) | |  |
|  |  |  | |  | |  | |  | |  | |  | |  | |  | |  | |  | |  | |  | |

†: respondents that have consented to linkage; 95% CI: 95% confidence interval; SD: standard deviation; MAR: missing-at-random; MNAR: missing-not-at-random; ^CR^ continuum of resistance-based sensitivity analysis; ^*^ slight sensitivity analysis; ^**^ moderate sensitivity analysis; ^***^ extreme sensitivity analyses.

**Supplementary Table S3c**: Weekly alcohol consumption estimates in the 2003 Scottish Health Survey respondents† aged 20 to 64 years by sex and area deprivation quintile under a range of assumption about the missing data: socio-demographic based survey weights; MAR; MNAR.

|  |  | Survey-weighted estimates among respondents | | | | MAR estimates in adjusted sample | | | | MNAR^CR^ estimates in adjusted sample | | | | MNAR^*^ estimates in adjusted sample | | | | MNAR^**^ estimates in adjusted sample | | | | MNAR^***^ estimates in adjusted sample | | | |
| --- | --- | --- | --- | --- | --- | --- | --- | --- | --- | --- | --- | --- | --- | --- | --- | --- | --- | --- | --- | --- | --- | --- | --- | --- | --- |
|  |  |  |  |  |  |  |  |  |  |  |  |  |  |  |  |  |  |  |  |  |  |  |  |  |  |
| Quintile of deprivation | N | Mean | (95% CI) | | Mean | | (95% CI) | | Mean | | (95% CI) | | Mean | | (95% CI) | | Mean | | (95% CI) | | Mean | | (95% CI) | |  |
| *Males* |  |  |  | |  | |  | |  | |  | |  | |  | |  | |  | |  | |  | |  |
| Least deprived | 484 | 23.1 | (20.9 - 25.3) | | 22.5 | | (19.3 - 25.7) | | 23.9 | | (20.7 - 27.1) | | 23.2 | | (20.1 - 26.4) | | 24.7 | | (21.2 - 28.1) | | 26.1 | | (22.0 - 30.2) | |  |
| 2 | 532 | 21.4 | (19.2 - 23.6) | | 20.0 | | (16.4 - 23.7) | | 21.9 | | (18.2 - 25.6) | | 21.4 | | (17.6 - 25.1) | | 24.1 | | (19.8 - 28.3) | | 26.8 | | (21.6 - 31.9) | |  |
| 3 | 500 | 21.9 | (18.8 - 25.0) | | 22.8 | | (18.8 - 26.9) | | 24.9 | | (20.6 - 29.1) | | 24.3 | | (20.0 - 28.7) | | 27.3 | | (22.1 - 32.5) | | 30.3 | | (23.9 - 36.7) | |  |
| 4 | 457 | 20.0 | (17.6 - 22.5) | | 20.2 | | (17.4 - 23.0) | | 22.9 | | (20.0 - 25.9) | | 22.8 | | (19.7 - 25.9) | | 28.0 | | (23.6 - 32.3) | | 33.1 | | (27.1 - 39.2) | |  |
| Most deprived | 380 | 22.5 | (17.7 - 27.3) | | 26.5 | | (18.6 - 34.4) | | 31.2 | | (23.0 - 39.4) | | 31.6 | | (23.3 - 40.0) | | 41.9 | | (32.2 - 51.6) | | 52.1 | | (40.5 - 63.8) | |  |
| *All quintiles* | 2,353 | 21.8 | (20.5 - 23.1) | | 22.4 | | (20.3 - 24.4) | | 24.9 | | (22.8 - 27.0) | | 24.6 | | (22.4 - 26.7) | | 28.9 | | (26.4 - 31.5) | | 33.3 | | (30.1 - 36.5) | |  |
|  |  |  |  | |  | |  | |  | |  | |  | |  | |  | |  | |  | |  | |  |
| *Females* |  |  |  | |  | |  | |  | |  | |  | |  | |  | |  | |  | |  | |  |
| Least deprived | 603 | 12.5 | (11.4 - 13.5) | | 12.9 | | (11.2 - 14.5) | | 13.5 | | (11.8 - 15.1) | | 13.0 | | (11.3 - 14.6) | | 13.2 | | (11.5 - 14.8) | | 13.4 | | (11.6 - 15.1) | |  |
| 2 | 666 | 12.7 | (10.3 - 15.1) | | 12.2 | | (9.4 - 15.0) | | 12.8 | | (10.0 - 15.6) | | 12.3 | | (9.4 - 15.1) | | 12.4 | | (9.6 - 15.3) | | 12.6 | | (9.7 - 15.5) | |  |
| 3 | 631 | 9.7 | (8.6 - 10.8) | | 9.6 | | (8.1 - 11.2) | | 10.3 | | (8.8 - 11.9) | | 9.8 | | (8.2 - 11.4) | | 10.1 | | (8.5 - 11.7) | | 10.4 | | (8.7 - 12.2) | |  |
| 4 | 586 | 9.5 | (8.3 - 10.8) | | 9.4 | | (7.7 - 11.1) | | 10.1 | | (8.4 - 11.8) | | 9.7 | | (8.0 - 11.4) | | 10.2 | | (8.4 - 12.1) | | 10.8 | | (8.8 - 12.8) | |  |
| Most deprived | 542 | 9.4 | (7.8 - 11.0) | | 9.7 | | (7.5 - 11.9) | | 10.5 | | (8.3 - 12.7) | | 10.2 | | (8.0 - 12.5) | | 11.2 | | (8.8 - 13.7) | | 12.3 | | (9.6 - 14.9) | |  |
| *All quintiles* | 3,028 | 10.8 | (10.1 - 11.6) | | 10.8 | | (9.8 - 11.7) | | 11.5 | | (10.5 - 12.4) | | 11.0 | | (10.0 - 12.0) | | 11.5 | | (10.5 - 12.5) | | 11.9 | | (10.8 - 13.0) | |  |
|  |  |  | |  | |  | |  | |  | |  | |  | |  | |  | |  | |  | |  | |

†: respondents that have consented to linkage; 95% CI: 95% confidence interval; SD: standard deviation; MAR: missing-at-random; MNAR: missing-not-at-random; ^CR^ continuum of resistance-based sensitivity analysis; ^*^ slight sensitivity analysis; ^**^ moderate sensitivity analysis; ^***^ extreme sensitivity analyses.

**Supplementary Table S3d**: Weekly alcohol consumption estimates in the 2008-10 Scottish Health Survey respondents† aged 20 to 64 years by sex and area deprivation quintile under a range of assumption about the missing data: socio-demographic based survey weights; MAR; MNAR.

|  |  | Survey-weighted estimates among respondents | | | | MAR estimates in adjusted sample | | | | MNAR^CR^ estimates in adjusted sample | | | | MNAR^*^ estimates in adjusted sample | | | | MNAR^**^ estimates in adjusted sample | | | | MNAR^***^ estimates in adjusted sample | | | |
| --- | --- | --- | --- | --- | --- | --- | --- | --- | --- | --- | --- | --- | --- | --- | --- | --- | --- | --- | --- | --- | --- | --- | --- | --- | --- |
|  |  |  |  |  |  |  |  |  |  |  |  |  |  |  |  |  |  |  |  |  |  |  |  |  |  |
| Quintile of deprivation | N | Mean | (95% CI) | | Mean | | (95% CI) | | Mean | | (95% CI) | | Mean | | (95% CI) | | Mean | | (95% CI) | | Mean | | (95% CI) | |  |
| *Males* |  |  |  | |  | |  | |  | |  | |  | |  | |  | |  | |  | |  | |  |
| Least deprived | 1,054 | 17.6 | (16.3 - 18.8) | | 17.3 | | (16.1 - 18.4) | | 18.2 | | (17.1 - 19.3) | | 17.3 | | (16.1 - 18.4) | | 17.3 | | (16.2 - 18.4) | | 17.3 | | (16.2 - 18.5) | |  |
| 2 | 991 | 17.6 | (16.3 - 18.9) | | 17.2 | | (16.0 - 18.5) | | 18.2 | | (16.9 - 19.4) | | 17.3 | | (16.0 - 18.6) | | 17.4 | | (16.1 - 18.8) | | 17.5 | | (16.1 - 19.0) | |  |
| 3 | 987 | 19.7 | (17.2 - 22.3) | | 19.3 | | (16.4 - 22.2) | | 20.5 | | (17.6 - 23.4) | | 19.7 | | (16.8 - 22.7) | | 20.6 | | (17.3 - 23.9) | | 21.4 | | (17.5 - 25.3) | |  |
| 4 | 1,097 | 17.6 | (16.1 - 19.0) | | 18.0 | | (16.6 - 19.5) | | 19.8 | | (18.2 - 21.4) | | 19.4 | | (17.8 - 21.1) | | 22.2 | | (19.9 - 24.6) | | 25.1 | | (21.8 - 28.3) | |  |
| Most deprived | 883 | 21.4 | (19.1 - 23.6) | | 24.3 | | (20.9 - 27.7) | | 27.4 | | (23.8 - 30.9) | | 27.6 | | (24.0 - 31.2) | | 34.3 | | (29.9 - 38.7) | | 40.9 | | (35.4 - 46.4) | |  |
| *All quintiles* | 5,012 | 18.8 | (17.9 - 19.6) | | 19.1 | | (18.2 - 20.1) | | 20.7 | | (19.7 - 21.7) | | 20.1 | | (19.1 - 21.1) | | 22.1 | | (20.9 - 23.3) | | 24.1 | | (22.6 - 25.6) | |  |
|  |  |  |  | |  | |  | |  | |  | |  | |  | |  | |  | |  | |  | |  |
| *Females* |  |  |  | |  | |  | |  | |  | |  | |  | |  | |  | |  | |  | |  |
| Least deprived | 1,544 | 10.3 | (9.4 - 11.2) | | 10.1 | | (9.3 - 10.9) | | 10.7 | | (9.9 - 11.4) | | 10.1 | | (9.3 - 10.9) | | 10.1 | | (9.3 - 10.9) | | 10.1 | | (9.3 - 10.9) | |  |
| 2 | 1,332 | 8.8 | (8.1 - 9.4) | | 8.8 | | (8.1 - 9.4) | | 9.3 | | (8.7 - 10.0) | | 8.8 | | (8.1 - 9.4) | | 8.8 | | (8.1 - 9.4) | | 8.8 | | (8.1 - 9.5) | |  |
| 3 | 1,292 | 8.9 | (8.2 - 9.7) | | 8.9 | | (7.9 - 9.9) | | 9.5 | | (8.5 - 10.5) | | 8.9 | | (7.9 - 9.9) | | 9.0 | | (7.9 - 10.0) | | 9.0 | | (7.9 - 10.1) | |  |
| 4 | 1,399 | 7.8 | (7.1 - 8.5) | | 7.9 | | (7.1 - 8.6) | | 8.4 | | (7.7 - 9.2) | | 8.0 | | (7.3 - 8.8) | | 8.3 | | (7.5 - 9.1) | | 8.6 | | (7.7 - 9.6) | |  |
| Most deprived | 1,155 | 8.4 | (7.5 - 9.2) | | 8.7 | | (7.6 - 9.7) | | 9.2 | | (8.2 - 10.2) | | 8.9 | | (7.9 - 10.0) | | 9.5 | | (8.4 - 10.6) | | 10.1 | | (8.9 - 11.3) | |  |
| *All quintiles* | 6,722 | 8.8 | (8.5 - 9.1) | | 8.8 | | (8.5 - 9.2) | | 9.4 | | (9.0 - 9.8) | | 8.9 | | (8.5 - 9.3) | | 9.1 | | (8.7 - 9.5) | | 9.3 | | (8.9 - 9.7) | |  |
|  |  |  | |  | |  | |  | |  | |  | |  | |  | |  | |  | |  | |  | |

†: respondents that have consented to linkage; 95% CI: 95% confidence interval; SD: standard deviation; MAR: missing-at-random; MNAR: missing-not-at-random; ^CR^ continuum of resistance-based sensitivity analysis; ^*^ slight sensitivity analysis; ^**^ moderate sensitivity analysis; ^***^ extreme sensitivity analyses.

**Supplementary Table S4a**: Weekly alcohol consumption estimates for individuals aged 20-to-64-years in 1995 by sex and area deprivation quintile calibrated to per capita estimates.

|  |  | |  |  |  | |  | |  | | |
| --- | --- | --- | --- | --- | --- | --- | --- | --- | --- | --- | --- |
| Quintile of deprivation | Calibrated | | Calibrated^CR^ | | Calibrated^*^ | | Calibrated^**^ | | Calibrated^***^ | | |
|  |  | |  |  |  | |  | |  |  | |
|  | Mean | *SD* | Mean | *SD* | Mean | *SD* | Mean | *SD* | Mean | *SD* | |
|  |  |  |  |  |  |  |  |  |  |  | |
|  |  |  |  |  | *Males* |  |  |  |  |  | |
|  |  |  |  |  |  |  |  |  |  |  | |
| Least deprived | 30.1 | *29.3* | 29.7 | *27.6* | 28.8 | *28.0* | 26.7 | *26.4* | 24.9 | *25.2* | |
| 2 | 31.6 | *34.6* | 31.6 | *32.7* | 31.3 | *34.7* | 30.8 | *35.8* | 30.4 | *36.8* | |
| 3 | 30.3 | *36.8* | 30.3 | *34.7* | 30.5 | *36.6* | 30.8 | *37.3* | 31.1 | *38.1* | |
| 4 | 34.0 | *40.5* | 34.9 | *38.2* | 35.5 | *42.8* | 38.0 | *50.3* | 40.0 | *57.1* | |
| Most deprived | 44.3 | *57.7* | 45.0 | *54.5* | 46.3 | *62.6* | 49.6 | *74.0* | 52.3 | *84.2* | |
| *All quintiles* | 33.8 | *39.3* | 34.0 | *37.1* | 34.1 | *40.4* | 34.7 | *43.9* | 35.1 | *47.0* |  |
|  |  |  |  |  |  |  |  |  |  |  |  |
|  |  |  |  |  | *Femaless* |  |  |  |  |  | |
|  |  |  |  |  |  |  |  |  |  |  | |
| Least deprived | 12.0 | *13.5* | 11.7 | *14.7* | 11.4 | *12.9* | 10.4 | *12.0* | 9.6 | *11.4* | |
| 2 | 9.4 | *11.6* | 9.2 | *11.3* | 9.0 | *11.2* | 8.5 | *10.8* | 8.1 | *10.5* | |
| 3 | 8.7 | *11.1* | 8.6 | *11.2* | 8.4 | *10.7* | 7.8 | *10.2* | 7.4 | *9.8* | |
| 4 | 10.7 | *20.3* | 10.5 | *13.0* | 10.4 | *19.5* | 10.0 | *18.4* | 9.7 | *17.6* | |
| Most deprived | 10.3 | *15.4* | 9.8 | *14.4* | 10.2 | *15.2* | 10.0 | *15.2* | 9.9 | *15.3* | |
| *All quintiles* | 10.2 | *14.3* | 10.0 | *13.5* | 9.9 | *13.8* | 9.4 | *13.3* | 8.9 | *12.8* | |
|  |  |  |  |  |  |  |  |  |  |  | |
|  |  |  |  |  |  |  |  |  |  |  | |

SD: standard deviation; ^CR^ continuum of resistance-based sensitivity analysis; ^*^ slight sensitivity analysis; ^**^ moderate sensitivity analysis; ^***^ extreme sensitivity analyses; Calibrated: calibrated to retail data.

**Supplementary Table S4b**: Weekly alcohol consumption estimates for individuals aged 20-to-64-years in 1998 by sex and area deprivation quintile calibrated to per capita estimates.

|  |  | |  |  |  | |  | |  | | |
| --- | --- | --- | --- | --- | --- | --- | --- | --- | --- | --- | --- |
| Quintile of deprivation | Calibrated | | Calibrated^CR^ | | Calibrated^*^ | | Calibrated^**^ | | Calibrated^***^ | | |
|  |  | |  |  |  | |  | |  |  | |
|  | Mean | *SD* | Mean | *SD* | Mean | *SD* | Mean | *SD* | Mean | *SD* | |
|  |  |  |  |  |  |  |  |  |  |  | |
|  |  |  |  |  | *Males* |  |  |  |  |  | |
|  |  |  |  |  |  |  |  |  |  |  | |
| Least deprived | 31.8 | *33.8* | 31.5 | *35.5* | 30.9 | *33.0* | 29.2 | *32.7* | 27.9 | *32.6* | |
| 2 | 33.2 | *37.4* | 33.3 | *36.9* | 33.0 | *37.9* | 32.6 | *39.3* | 32.2 | *40.6* | |
| 3 | 31.0 | *37.2* | 31.3 | *31.1* | 31.6 | *38.4* | 32.8 | *42.5* | 33.7 | *46.1* | |
| 4 | 35.3 | *42.2* | 36.2 | *38.7* | 36.7 | *45.4* | 39.0 | *52.7* | 41.0 | *59.2* | |
| Most deprived | 43.1 | *52.9* | 43.3 | *50.9* | 44.2 | *56.9* | 46.0 | *67.6* | 47.5 | *77.4* | |
| *All quintiles* | 34.6 | *40.4* | 34.8 | *38.2* | 34.9 | *41.8* | 35.4 | *46.1* | 35.8 | *49.9* |  |
|  |  |  |  |  |  |  |  |  |  |  |  |
|  |  |  |  |  | *Femaless* |  |  |  |  |  | |
|  |  |  |  |  |  |  |  |  |  |  | |
| Least deprived | 11.9 | *15.8* | 11.8 | *13.9* | 11.4 | *15.1* | 10.5 | *14.0* | 9.8 | *13.1* | |
| 2 | 12.3 | *14.5* | 12.2 | *13.8* | 11.9 | *14.2* | 11.1 | *13.8* | 10.5 | *13.6* | |
| 3 | 11.6 | *15.7* | 11.3 | *14.3* | 11.4 | *15.2* | 11.0 | *14.8* | 10.7 | *14.6* | |
| 4 | 10.7 | *15.3* | 10.5 | *14.2* | 10.6 | *15.1* | 10.3 | *15.5* | 10.1 | *15.8* | |
| Most deprived | 11.7 | *17.0* | 11.4 | *15.0* | 11.7 | *16.8* | 11.5 | *17.0* | 11.5 | *17.2* | |
| *All quintiles* | 11.7 | *15.6* | 11.5 | *14.3* | 11.4 | *15.2* | 10.9 | *14.9* | 10.5 | *14.7* | |
|  |  |  |  |  |  |  |  |  |  |  | |
|  |  |  |  |  |  |  |  |  |  |  | |

SD: standard deviation; ^CR^ continuum of resistance-based sensitivity analysis; ^*^ slight sensitivity analysis; ^**^ moderate sensitivity analysis; ^***^ extreme sensitivity analyses; Calibrated: calibrated to retail data.

**Supplementary Table S4c**: Weekly alcohol consumption estimates for individuals aged 20-to-64-years in 2003 by sex and area deprivation quintile calibrated to per capita estimates.

|  |  | |  |  |  | |  | |  | | |
| --- | --- | --- | --- | --- | --- | --- | --- | --- | --- | --- | --- |
| Quintile of deprivation | Calibrated | | Calibrated^CR^ | | Calibrated^*^ | | Calibrated^**^ | | Calibrated^***^ | | |
|  |  | |  |  |  | |  | |  |  | |
|  | Mean | *SD* | Mean | *SD* | Mean | *SD* | Mean | *SD* | Mean | *SD* | |
|  |  |  |  |  |  |  |  |  |  |  | |
|  |  |  |  |  | *Males* |  |  |  |  |  | |
|  |  |  |  |  |  |  |  |  |  |  | |
| Least deprived | 33.4 | *35.6* | 32.4 | *30.7* | 32.1 | *34.3* | 30.1 | *32.6* | 28.5 | *31.4* | |
| 2 | 29.8 | *32.1* | 29.7 | *29.2* | 29.6 | *33.2* | 29.4 | *36.8* | 29.2 | *40.0* | |
| 3 | 33.9 | *38.4* | 33.7 | *37.4* | 33.7 | *38.7* | 33.3 | *39.8* | 33.0 | *40.8* | |
| 4 | 30.0 | *33.4* | 31.1 | *31.7* | 31.6 | *36.6* | 34.1 | *46.0* | 36.1 | *54.2* | |
| Most deprived | 39.4 | *69.6* | 42.3 | *50.4* | 43.8 | *72.8* | 51.1 | *88.2* | 56.9 | *101.7* | |
| *All quintiles* | 33.2 | *41.1* | 33.7 | *35.7* | 34.0 | *42.3* | 35.3 | *47.2* | 36.4 | *51.5* |  |
|  |  |  |  |  |  |  |  |  |  |  |  |
|  |  |  |  |  | *Femaless* |  |  |  |  |  | |
|  |  |  |  |  |  |  |  |  |  |  | |
| Least deprived | 19.1 | *21.9* | 18.3 | *18.5* | 18.0 | *20.4* | 16.1 | *18.4* | 14.6 | *16.8* | |
| 2 | 18.0 | *20.3* | 17.3 | *27.4* | 17.0 | *19.0* | 15.2 | *17.2* | 13.8 | *15.7* | |
| 3 | 14.3 | *19.2* | 14.0 | *18.7* | 13.5 | *18.2* | 12.3 | *16.6* | 11.4 | *15.5* | |
| 4 | 14.0 | *19.3* | 13.7 | *18.2* | 13.4 | *18.6* | 12.5 | *17.7* | 11.8 | *17.0* | |
| Most deprived | 14.4 | *19.6* | 14.2 | *17.0* | 14.2 | *19.2* | 13.7 | *18.8* | 13.4 | *18.5* | |
| *All quintiles* | 16.0 | *20.4* | 15.5 | *20.2* | 15.2 | *19.4* | 14.0 | *18.0* | 13.0 | *16.9* | |
|  |  |  |  |  |  |  |  |  |  |  | |
|  |  |  |  |  |  |  |  |  |  |  | |

SD: standard deviation; ^CR^ continuum of resistance-based sensitivity analysis; ^*^ slight sensitivity analysis; ^**^ moderate sensitivity analysis; ^***^ extreme sensitivity analyses; Calibrated: calibrated to retail data.

**Supplementary Table S4d**: Weekly alcohol consumption estimates for individuals aged 20-to-64-years in 2008-10 by sex and area deprivation quintile calibrated to per capita estimates.

|  |  | |  |  |  | |  | |  | | |
| --- | --- | --- | --- | --- | --- | --- | --- | --- | --- | --- | --- |
| Quintile of deprivation | Calibrated | | Calibrated^CR^ | | Calibrated^*^ | | Calibrated^**^ | | Calibrated^***^ | | |
|  |  | |  |  |  | |  | |  |  | |
|  | Mean | *SD* | Mean | *SD* | Mean | *SD* | Mean | *SD* | Mean | *SD* | |
|  |  |  |  |  |  |  |  |  |  |  | |
|  |  |  |  |  | *Males* |  |  |  |  |  | |
|  |  |  |  |  |  |  |  |  |  |  | |
| Least deprived | 30.2 | *30.0* | 29.6 | *31.1* | 29.1 | *28.9* | 27.1 | *26.9* | 25.4 | *25.2* | |
| 2 | 30.2 | *35.3* | 29.5 | *31.7* | 29.1 | *34.3* | 27.3 | *26.9* | 25.7 | *31.0* | |
| 3 | 33.7 | *41.6* | 33.3 | *43.4* | 33.2 | *41.5* | 32.2 | *41.7* | 31.4 | *41.9* | |
| 4 | 31.5 | *40.3* | 32.2 | *37.7* | 32.7 | *43.5* | 34.9 | *41.5* | 36.7 | *57.2* | |
| Most deprived | 42.5 | *54.9* | 44.5 | *58.4* | 46.5 | *43.5* | 53.7 | *41.5* | 60.0 | *100.7* | |
| *All quintiles* | 33.5 | *39.9* | 33.6 | *40.1* | 33.9 | *41.5* | 34.6 | *45.3* | 35.3 | *48.8* |  |
|  |  |  |  |  |  |  |  |  |  |  |  |
|  |  |  |  |  | *Femaless* |  |  |  |  |  | |
|  |  |  |  |  |  |  |  |  |  |  | |
| Least deprived | 17.6 | *20.1* | 17.3 | *19.4* | 17.0 | *19.4* | 15.8 | *18.1* | 14.8 | *16.9* | |
| 2 | 15.3 | *20.4* | 15.2 | *18.4* | 14.7 | *19.7* | 13.7 | *18.4* | 12.9 | *17.3* | |
| 3 | 15.6 | *20.8* | 15.5 | *20.2* | 15.0 | *20.1* | 14.1 | *19.0* | 13.2 | *18.0* | |
| 4 | 13.7 | *20.0* | 13.7 | *19.8* | 13.5 | *19.5* | 13.0 | *19.0* | 12.6 | *18.5* | |
| Most deprived | 15.1 | *21.4* | 15.0 | *20.8* | 15.1 | *21.3* | 14.9 | *21.5* | 14.8 | *21.8* | |
| *All quintiles* | 15.5 | *20.7* | 15.3 | *19.8* | 15.0 | *20.2* | 14.3 | *19.3* | 13.7 | *18.6* | |
|  |  |  |  |  |  |  |  |  |  |  | |
|  |  |  |  |  |  |  |  |  |  |  | |

SD: standard deviation; ^CR^ continuum of resistance-based sensitivity analysis; ^*^ slight sensitivity analysis; ^**^ moderate sensitivity analysis; ^***^ extreme sensitivity analyses; Calibrated: calibrated to retail data.

**Supplementary Table S5**: Potential problem-drinker prevalence estimates (among current drinkers) in the Scottish Health Survey respondents† and in the “full sample|” by survey year, sex and area deprivation quintile

|  | Males | | | | Females | | | | | |
| --- | --- | --- | --- | --- | --- | --- | --- | --- | --- | --- |
|  | Survey-weighted | | MAR | | Survey-weighted | | | MAR | |  |
| Quintile of deprivation | % | (CI) | % | (CI) | % | | (CI) | % | (CI) |  |
|  |  |  |  |  | |  |  |  |  |  |
|  |  |  |  | *1998* | |  |  |  |  |  |
|  |  |  |  |  | |  |  |  |  |  |
| Least deprived | 10.1 | (7.1 - 13.1) | 10.2 | (7.4 - 13.0) | | 4.9 | (3.0 - 6.8) | 5.0 | (3.1 - 6.9) |  |
| 2 | 8.4 | (5.6 - 11.2) | 8.7 | (6.1 - 11.2) | | 4.8 | (2.8 - 6.8) | 4.8 | (2.9 - 6.7) |  |
| 3 | 10.6 | (7.7 - 13.5) | 11.4 | (8.5 - 14.2) | | 3.9 | (2.2 - 5.6) | 4.5 | (2.7 - 6.3) |  |
| 4 | 12.7 | (9.3 - 16.1) | 14.3 | (10.8 - 17.8) | | 5.0 | (3.1 - 6.8) | 5.5 | (3.5 - 7.5) |  |
| Most deprived | 20.3 | (16.3 - 24.3) | 21.7 | (17.8 - 25.6) | | 6.6 | (4.2 - 9.0) | 7.7 | (5.3 - 10.1) |  |
| *All quintiles* | 12.3 | (10.9 - 13.8) | 12.9 | (11.5 - 14.3) | | 5.0 | (4.1 - 5.8) | 5.4 | (4.5 - 6.3) |  |
|  |  |  |  |  | |  |  |  |  |  |
|  |  |  |  | *2003* | |  |  |  |  |  |
|  |  |  |  |  | |  |  |  |  |  |
| Least deprived | 10.9 | (7.7 - 14.1) | 9.9 | (7.1 - 12.8) | | 6.1 | (4.0 - 8.2) | 6.4 | (4.3 - 8.4) |  |
| 2 | 8.6 | (6.0 - 11.2) | 10.0 | (7.1 - 13.0) | | 5.6 | (3.5 - 7.6) | 5.9 | (3.9 - 7.8) |  |
| 3 | 11.1 | (8.0 - 14.2) | 12.4 | (9.3 - 15.5) | | 3.8 | (2.3 - 5.4) | 3.8 | (2.2 - 5.5) |  |
| 4 | 13.6 | (10.1 - 17.0) | 14.5 | (11.1 - 18.0) | | 5.7 | (3.6 - 7.8) | 5.8 | (3.7 - 7.8) |  |
| Most deprived | 16.8 | (12.9 - 20.7) | 18.8 | (14.7 - 23.0) | | 8.8 | (6.2 - 11.4) | 9.2 | (6.6 - 11.9) |  |
| *All quintiles* | 12.8 | (11.2 - 14.3) | 13.3 | (11.8 - 14.7) | | 6.7 | (5.6 - 7.7) | 6.6 | (5.6 - 7.7) |  |
|  |  |  |  |  | |  |  |  |  |  |
|  |  |  |  | *2008/10* | |  |  |  |  |  |
|  |  |  |  |  | |  |  |  |  |  |
| Least deprived | 11.1 | (8.8 - 13.5) | 11.4 | (9.5 - 13.3) | | 7.4 | (5.7 - 9.1) | 7.1 | (5.7 - 8.5) |  |
| 2 | 12.1 | (9.6 - 14.6) | 11.4 | (9.4 - 13.4) | | 5.7 | (4.2 - 7.1) | 5.5 | (4.4 - 6.7) |  |
| 3 | 13.3 | (10.6 - 16.0) | 13.2 | (11.0 - 15.5) | | 9.2 | (7.4 - 11.0) | 9.4 | (7.5 - 11.2) |  |
| 4 | 14.5 | (12.0 - 17.0) | 16.2 | (13.8 - 18.5) | | 7.8 | (6.2 - 9.4) | 8.2 | (6.6 - 9.9) |  |
| Most deprived | 16.2 | (13.7 - 18.6) | 18.4 | (15.9 - 20.9) | | 9.7 | (8.0 - 11.4) | 10.3 | (8.7 - 12.0) |  |
| *All quintiles* | 14.8 | (13.6 - 16.0) | 14.7 | (13.6 - 15.8) | | 9.1 | (8.3 - 10.0) | 8.7 | (8.0 - 9.4) |  |
|  |  |  |  |  | |  |  |  |  |  |

† Respondents that have consented to linkage; MAR: missing-at-random

**Supplementary Table S6**: Binge-drinking prevalence estimates (among those who drank in last seven days) in the Scottish Health Survey respondents† and in the adjusted sample by survey year, sex and area deprivation quintile

|  | Males | | | | Females | | | | | |
| --- | --- | --- | --- | --- | --- | --- | --- | --- | --- | --- |
|  | Survey-weighted | | MAR | | Survey-weighted | | | MAR | |  |
| Quintile of deprivation | % | (CI) | % | (CI) | % | | (CI) | % | (CI) |  |
|  |  |  |  |  | |  |  |  |  |  |
|  |  |  |  | *1998* | |  |  |  |  |  |
|  |  |  |  |  | |  |  |  |  |  |
| Least deprived | 31.2 | (26.3 - 36.1) | 30.9 | (25.1 - 36.8) | | 16.6 | (12.8 - 20.4) | 16.0 | (11.7 - 20.3) |  |
| 2 | 37.4 | (32.3 - 42.5) | 36.7 | (31.8 - 41.7) | | 14.9 | (11.2 - 18.5) | 14.5 | (9.9 - 19.1) |  |
| 3 | 37.9 | (32.9 - 42.8) | 38.2 | (33.1 - 43.4) | | 17.0 | (13.4 - 20.6) | 18.3 | (13.9 - 22.6) |  |
| 4 | 44.2 | (38.9 - 49.6) | 44.0 | (38.6 - 49.3) | | 21.2 | (17.1 - 25.4) | 20.9 | (16.1 - 25.7) |  |
| Most deprived | 49.3 | (43.9 - 54.6) | 49.9 | (44.7 - 55.1) | | 25.7 | (20.7 - 30.8) | 26.7 | (21.1 - 32.2) |  |
| *All quintiles* | 39.7 | (37.4 - 42.0) | 39.2 | (36.6 - 41.7) | | 18.8 | (18.6-19.0) | 18.6 | (16.6 - 20.7) |  |
|  |  |  |  |  | |  |  |  |  |  |
|  |  |  |  | *2003* | |  |  |  |  |  |
|  |  |  |  |  | |  |  |  |  |  |
| Least deprived | 27.2 | (22.5 - 32.0) | 27.5 | (21.3 - 33.6) | | 18.4 | (14.4 - 22.5) | 17.4 | (12.7 - 22.1) |  |
| 2 | 29.8 | (24.9 - 34.8) | 30.1 | (24.5 - 35.8) | | 15.4 | (11.7 - 19.1) | 15.7 | (11.6 - 19.9) |  |
| 3 | 34.1 | (28.6 - 39.6) | 34.0 | (28.2 - 39.8) | | 19.3 | (14.9 - 23.8) | 19.4 | (14.2 - 24.6) |  |
| 4 | 40.1 | (34.3 - 46.0) | 39.7 | (32.6 - 46.8) | | 23.3 | (18.5 - 28.2) | 21.1 | (15.3 - 26.9) |  |
| Most deprived | 44.5 | (37.8 - 51.2) | 44.0 | (38.1 - 49.9) | | 33.9 | (27.9 - 39.9) | 34.5 | (28.0 - 40.9) |  |
| *All quintiles* | 34.2 | (31.7 - 36.6) | 34.3 | (31.5 - 37.0) | | 21.1 | (19.1 - 23.2) | 20.9 | (18.5 - 23.2) |  |
|  |  |  |  |  | |  |  |  |  |  |
|  |  |  |  | *2008/10* | |  |  |  |  |  |
|  |  |  |  |  | |  |  |  |  |  |
| Least deprived | 38.0 | (34.1 - 41.9) | 38.8 | (35.3 - 42.3) | | 28.8 | (25.5 - 32.2) | 31.5 | (28.4 - 34.6) |  |
| 2 | 41.4 | (37.4 - 45.4) | 40.8 | (37.3 - 44.2) | | 30.4 | (27.0 - 33.8) | 31.8 | (28.5 - 35.0) |  |
| 3 | 42.4 | (38.1 - 46.7) | 42.4 | (38.8 - 46.0) | | 35.1 | (31.3 - 38.8) | 37.6 | (34.1 - 41.0) |  |
| 4 | 47.8 | (43.6 - 52.1) | 48.9 | (45.1 - 52.6) | | 34.3 | (30.5 - 38.0) | 36.9 | (33.4 - 40.5) |  |
| Most deprived | 47.2 | (43.1 - 51.4) | 47.9 | (44.0 - 51.8) | | 42.6 | (38.9 - 46.3) | 43.7 | (40.1 - 47.3) |  |
| *All quintiles* | 43.2 | (41.3 - 45.0) | 43.3 | (41.7 - 44.9) | | 33.9 | (32.3 - 35.5) | 35.7 | (34.1 - 37.2) |  |

† Respondents that have consented to linkage; MAR: missing-at-random

**Supplementary Table S7**: Current non-drinker prevalence estimates in the Scottish Health Survey respondents† and in the “full sample|” by survey year, sex and area deprivation quintile

|  | Males | | | | Females | | | | | | |  |  |
| --- | --- | --- | --- | --- | --- | --- | --- | --- | --- | --- | --- | --- | --- |
|  | Survey-weighted | | MAR | | Survey-weighted | | | MAR | | | |  |  |
| Quintile of deprivation | % | (CI) | % | (CI) | % | | (CI) | % | | (CI) | |  |  |
|  |  |  |  |  |  | |  |  | |  | |  |  |
|  |  |  |  |  | |  |  | |  | |  | |  |
|  |  |  |  | *1995* | |  |  | |  | |  | |  |
|  |  |  |  |  | |  |  | |  | |  | |  |
| Least deprived | 3.5 | (1.9 - 5.1) | 4.0 | (2.2 - 5.9) | | 7.9 | (5.6 - 10.3) | | 7.8 | | (5.6 - 9.9) | |  |
| 2 | 5.7 | (3.7 - 7.6) | 5.9 | (4.0 - 7.9) | | 10.8 | (8.3 - 13.2) | | 10.7 | | (8.3 - 13.1) | |  |
| 3 | 5.3 | (3.4 - 7.2) | 5.4 | (3.5 - 7.3) | | 10.4 | (8.0 - 12.8) | | 10.4 | | (8.1 - 12.8) | |  |
| 4 | 4.5 | (2.7 - 6.3) | 5.0 | (2.9 - 7.0) | | 9.9 | (7.4 - 12.4) | | 10.1 | | (7.5 - 12.7) | |  |
| Most deprived | 6.6 | (4.6 - 8.6) | 6.5 | (4.5 - 8.5) | | 10.5 | (8.3 - 12.7) | | 10.4 | | (8.2 - 12.6) | |  |
| *All quintiles* | 5.1 | (4.3 - 6.0) | 5.3 | (4.5 - 6.2) | | 9.9 | (8.8 - 11.0) | | 9.8 | | (8.8 - 10.9) | |  |
|  |  |  |  |  | |  |  | |  | |  | |  |
|  |  |  |  | *1998* | |  |  | |  | |  | |  |
|  |  |  |  |  | |  |  | |  | |  | |  |
| Least deprived | 4.4 | (2.6 - 6.2) | 5.0 | (3.0 - 7.0) | | 7.8 | (5.3 - 10.3) | | 7.6 | | (5.4 - 9.7) | |  |
| 2 | 4.7 | (2.9 - 6.5) | 5.1 | (3.2 - 7.0) | | 6.3 | (4.1 - 8.5) | | 6.4 | | (4.3 - 8.5) | |  |
| 3 | 6.9 | (4.8 - 9.0) | 7.0 | (4.9 - 9.1) | | 9.5 | (7.4 - 11.6) | | 9.5 | | (7.3 - 11.6) | |  |
| 4 | 6.4 | (3.9 - 9.0) | 6.5 | (4.1 - 8.9) | | 10.5 | (8.0 - 12.9) | | 10.9 | | (8.3 - 13.4) | |  |
| Most deprived | 7.5 | (5.1 - 9.8) | 7.9 | (5.5 - 10.3) | | 16.0 | (12.9 - 19.1) | | 15.7 | | (12.6 - 18.8) | |  |
| *All quintiles* | 6.0 | (5.0 - 6.9) | 6.2 | (5.3 - 7.2) | | 10.1 | (8.9 - 11.2) | | 9.8 | | (8.7 - 10.9) | |  |
|  |  |  |  |  | |  |  | |  | |  | |  |
|  |  |  |  | *2003* | |  |  | |  | |  | | |
|  |  |  |  |  | |  |  | |  | |  | | |
| Least deprived | 4.3 | (2.3 - 6.2) | 4.7 | (2.6 - 6.9) | | 7.6 | (5.4 - 9.8) | | 8.0 | | (5.8 - 10.2) | |  |
| 2 | 4.6 | (2.6 - 6.6) | 5.0 | (3.1 - 7.0) | | 8.1 | (5.9 - 10.3) | | 8.5 | | (6.2 - 10.8) | |  |
| 3 | 5.5 | (3.4 - 7.7) | 6.2 | (3.8 - 8.6) | | 8.8 | (6.0 - 11.6) | | 8.5 | | (6.1 - 11.0) | |  |
| 4 | 6.4 | (4.2 - 8.7) | 7.6 | (5.1 - 10.0) | | 13.1 | (10.2 - 16.0) | | 13.6 | | (10.9 - 16.4) | |  |
| Most deprived | 12.3 | (8.9 - 15.8) | 14.7 | (10.9 - 18.4) | | 14.6 | (11.5 - 17.6) | | 14.6 | | (11.5 - 17.7) | |  |
| *All quintiles* | 6.4 | (5.4 - 7.5) | 7.5 | (6.3 - 8.7) | | 10.3 | (9.2 - 11.5) | | 10.6 | | (9.5 - 11.8) | |  |
|  |  |  |  |  | |  |  | |  | |  | |  |
|  |  |  |  | *2008/10* | |  |  | |  | |  | |  |
|  |  |  |  |  | |  |  | |  | |  | |  |
| Least deprived | 5.8 | (4.0 - 7.7) | 5.9 | (4.3 - 7.5) | | 7.2 | (5.6 - 8.7) | | 7.2 | | (5.8 - 8.7) | |  |
| 2 | 7.2 | (5.4 - 8.9) | 7.2 | (5.6 - 8.8) | | 9.1 | (7.3 - 10.8) | | 9.0 | | (7.5 - 10.5) | |  |
| 3 | 9.3 | (7.2 - 11.3) | 9.6 | (7.8 - 11.4) | | 12.1 | (10.1 - 14.1) | | 12.5 | | (10.5 - 14.6) | |  |
| 4 | 11.5 | (9.3 - 13.7) | 11.6 | (9.7 - 13.5) | | 15.8 | (13.7 - 17.9) | | 15.9 | | (13.8 - 18.0) | |  |
| Most deprived | 13.3 | (11.0 - 15.6) | 12.8 | (10.6 - 15.0) | | 16.8 | (14.8 - 18.7) | | 16.0 | | (14.1 - 17.8) | |  |
| *All quintiles* | 9.5 | (8.6 - 10.4) | 9.3 | (8.5 - 10.2) | | 12.4 | (11.6 - 13.3) | | 12.2 | | (11.3 - 13.1) | |  |
|  |  |  |  |  | |  |  | |  | |  | |  |

† Respondents that have consented to linkage; MAR: missing-at-random

**References**

1. Box G. E., Cox D. R. An analysis of transformations, Journal of the Royal Statistical Society Series B (Methodological) 1964: 211-252.

2. Royston P., White I. R. Multiple imputation by chained equations (MICE): implementation in Stata, Journal of Statistical Software 2011: 45: 1-20.

3. Rubin D. B. Inference and Missing Data, Biometrika 1976: 63: 581-592.

4. Gray L., Gorman E., White I. R., Katikireddi S. V., McCartney G., Rutherford L. et al. Correcting for bias arising from survey non-response: A novel methodology with application of pattern mixture modelling using record-linked data, (under review).

5. World Health Organization. International guide for monitoring alcohol consumption and related harm, 2000.

6. Robinson M., Thorpe R., Beeston C., McCartney G. A Review of the Validity and Reliability of Alcohol Retail Sales Data for Monitoring Population Levels of Alcohol Consumption: A Scottish Perspective, Alcohol Alcohol 2012.

7. Rehm J., Kehoe T., Gmel G., Stinson F., Grant B. Statistical modeling of volume of alcohol exposure for epidemiological studies of population health: the US example, Popul Health Metr 2010: 8: 3.

8. Meier P. S., Meng Y., Holmes J., Baumberg B., Purshouse R., Hill-McManus D. et al. Adjusting for unrecorded consumption in survey and per capita sales data: quantification of impact on gender- and age-specific alcohol-attributable fractions for oral and pharyngeal cancers in Great Britain, Alcohol Alcohol 2013: 48: 241-249.

9. Beeston C, Reid G, Robinson M, Craig N, McCartney G, Graham L et al. Monitoring and Evaluating Scotland’s Alcohol Strategy. Third Annual Report. In: Scotland N. H., editor, Edinburgh; 2013.

10. Mittelhammer R. C. Mathematical Statistics for Economics and Business: Springer; 2013.

11. Stockwell T., Zhao J., Macdonald S. Who under-reports their alcohol consumption in telephone surveys and by how much? An application of the Yesterday Method in a national Canadian substance use survey, Addiction 2014: n/a-n/a.
